# Genome-Wide Analysis in Swine Associates Corneal Graft Rejection with Donor-Recipient Mismatches in Three Novel Histocompatibility Regions and One Locus Homologous to the Mouse H-3 Locus

**DOI:** 10.1371/journal.pone.0152155

**Published:** 2016-03-24

**Authors:** Susan Nicholls, Ricardo Pong-Wong, Louisa Mitchard, Ross Harley, Alan Archibald, Andrew Dick, Michael Bailey

**Affiliations:** 1 School of Clinical Sciences, University of Bristol, Bristol, United Kingdom; 2 Royal Dick School of Veterinary Studies, The Roslin Institute and R(D)SVS, University of Edinburgh, Edinburgh, United Kingdom; 3 School of Veterinary Sciences, University of Bristol, Bristol, United Kingdom; 4 School of Cellular and Molecular Medicine, University of Bristol, Bristol, United Kingdom; 5 National Institute for Health Research Biomedical Research Centre at Moorfields Eye Hospital NHS Foundation Trust and UCL Institute of Ophthalmology, London, United Kingdom; INSERM, FRANCE

## Abstract

In rodents, immune responses to minor histocompatibility antigens are the most important drivers of corneal graft rejection. However, this has not been confirmed in humans or in a large animal model and the genetic loci are poorly characterised, even in mice. The gene sequence data now available for a range of relevant species permits the use of genome-wide association (GWA) techniques to identify minor antigens associated with transplant rejection. We have used this technique in a pre-clinical model of corneal transplantation in semi-inbred NIH minipigs and Babraham swine to search for novel minor histocompatibility loci and to determine whether rodent findings have wider applicability. DNA from a cohort of MHC-matched and MHC-mismatched donors and recipients was analysed for single nucleotide polymorphisms (SNPs). The level of SNP homozygosity for each line was assessed. Genome-wide analysis of the association of SNP disparities with rejection was performed using log-likelihood ratios. Four genomic blocks containing four or more SNPs significantly linked to rejection were identified (on chromosomes 1, 4, 6 and 9), none at the location of the MHC. One block of 36 SNPs spanned a region that exhibits conservation of synteny with the mouse H-3 histocompatibility locus and contains the pig homologue of the mouse *Zfp106* gene, which encodes peptide epitopes known to mediate corneal graft rejection. The other three regions are novel minor histocompatibility loci. The results suggest that rejection can be predicted from SNP analysis prior to transplant in this model and that a similar GWA analysis is merited in humans.

## Introduction

The accumulation in recent years of increasingly detailed and accurate sequence data for the human genome and for the genomes of mammalian species used in medical and veterinary research has enabled powerful analytical methods to be employed to identify genetic loci accounting for pathological conditions. In the transplantation field such methods have, for example, identified minor histocompatibility antigens (mHags) governing the success of bone marrow transplantation [1], liver transplant rejection [2] and kidney allograft function [3], but have not yet been applied to corneal graft rejection. Genome-wide association (GWA) studies using variation data collected by the human 1000 Genomes Project is further facilitating the identification of clinically relevant mHags by eliminating the need for time-consuming T-cell epitope identification strategies [4].

Although the importance of mHags in the immune response to corneas have long been established in rodent models [5, 6], their identities, both genetic and protein, have remained obscure with the exception of the mouse H-3 locus on chromosome 2. The H-3 locus consists of two closely linked genes, H-3a and H-3b [7]. While the precise genomic location and protein epitopes of H-3b are still uncertain, H-3a epitopes are encoded by a transcription regulator, *Zfp106* [8]. Orthologues of the murine *Zfp106* gene have been identified in other species including human (*ZNF106*) and pig (*ZNF106 aka ZFP106*). Two CD8 T cell epitopes in the Zfp106 protein contribute to corneal graft rejection in the mouse [9], each resulting from a single nucleotide substitution.

We have recently developed a pre-clinical model of corneal graft rejection in the semi-inbred NIH minipig [10], in which clinical rejection resembles that in man more closely than do rodent models, while at the same time permitting the study of immunity to defined major histocompatibility MHC mismatches. Contrary to rodent data, rejection did not occur in donor-recipient combinations that were matched at MHC loci, and only in 60% of MHC-mismatched combinations. To explain this discrepancy, to test the power of GWA to identify mHags in the pig and to further evaluate the pig model in relation to rodents and humans, we undertook GWA analysis to identify single nucleotide polymorphisms associated with rejection. The analysis also revealed the level of inbreeding within each of the three pig lines used.

## Materials and Methods

### Corneal transplantation

Swine leukocyte antigen (SLA)^cc^ and SLA^dd^ lines of NIH minipig ([11] https://www.ebi.ac.uk/ipd/mhc/sla/haplotypes.html) and large White Babraham pigs [12] were obtained from the Institute of Animal Health, Compton UK (now known as the Pirbright Institute). The Babraham line has recently been officially designated as homozygous Lr-55.6 (J. Hammond, personal communication), but this has not yet been published or entered in the database and we refer to the line as SLA^bb^ for the purposes of this paper. Each line is homozygous at the MHC locus, but retains intra-line minor locus incompatibilities [11, 12]. All procedures received prior approved by the University of Bristol Ethics of Research Committee and were performed in strict accordance with UK Scientific Procedures legislation and Medical Research Council policy to minimize numbers of animals used in scientific research. Anaesthesia was performed and pain relief prescribed and supervised by a qualified veterinary anaesthetist. Corneal transplantation, perioperative procedures and outcome were previously described [10, 13]. Briefly, corneas from SLA^bb^ Babraham (n = 6) or SLA^dd^ line minipigs (n = 10) were transplanted to SLA^cc^ line minipig recipients, each combination providing both major and minor histocompatibility mismatches. A third group of SLA^cc^ minipig intra-line transplants (n = 5) yielded minor histocompatibility mismatches alone. Recipients were of both sexes, but X-Y mismatches were excluded.

### Extraction of DNA and SNP genotyping

Blood was collected at surgery into ethylenediaminetetraacetic acid anticoagulant. DNA was extracted from 200μl of blood using the NucleoSpin Blood kit (Macherey-Nagel, Germany) as per manufacturer's instructions, then stored at -20°C.

DNA samples were genotyped using the Illumina PorcineSNP60 chip [14] in the ARK-Genomics Centre for Comparative and Functional Genomics at The Roslin Institute (www.ark-genomics.org) (ARK-Genomics is now integrated into Edinburgh Genomics (http://genomics.ed.ac.uk). The locations of SNPs in the Sscrofa9 genome assembly were used in the analysis, but were updated during manuscript preparation to locations of the Sscrofa 10.2 assembly (Ensembl release 83).

### Genome-wide association analysis

A GWA analysis was performed to identify minor loci affecting outcome. The genotype match/mismatch between donor and recipients was defined assuming two different rejection models. Model 1, consistent with a T cell epitope model of rejection, took into account the direction of the mismatch at the particular locus: i.e. a heterozygous graft AB was mismatched with a recipient of homozygous AA or BB genotype, whereas a mismatch in the opposite direction, i.e. an AA or BB donor in an AB recipient was matched (graft versus host reactions being irrelevant in this model). Model 2 took no account of the direction of the mismatch, i.e. both AB into AA/BB and AA/BB into AB were considered mismatches.

Hence, for a given SNP, genotypes of donor and recipient were classified as matched or mismatched and the outcome of the graft as accepted or rejected, thus yielding four possible graft outcome groups: matched-accepted, matched-rejected, mismatched-accepted, mismatched-rejected. The association of the SNP with corneal transplant outcome could be tested using the likelihood ratio test (LRT), equal to:

$$LRT=\sum_{i=1,2}\sum_{j=1,2}O_{ij}\;\ln\left(O_{ij}/E_{ij}\right)$$

where, *O_ij_* and *E_ij_* are the observed and expected counts for each of the possible groups (*i* = accepted or rejected; and *j* = matched, mismatched). Under the null hypothesis, the LRT follows a χ^2^ distribution with one degree of freedom [15]. Because of multiple testing, the empirical distribution of the null hypothesis was calculated using permutation analysis [16] where the 5% genome-wide significant threshold was obtained using 100,000 permutations.

## Results

### Genetic homozygosity within pig lines

**Fig 1:**
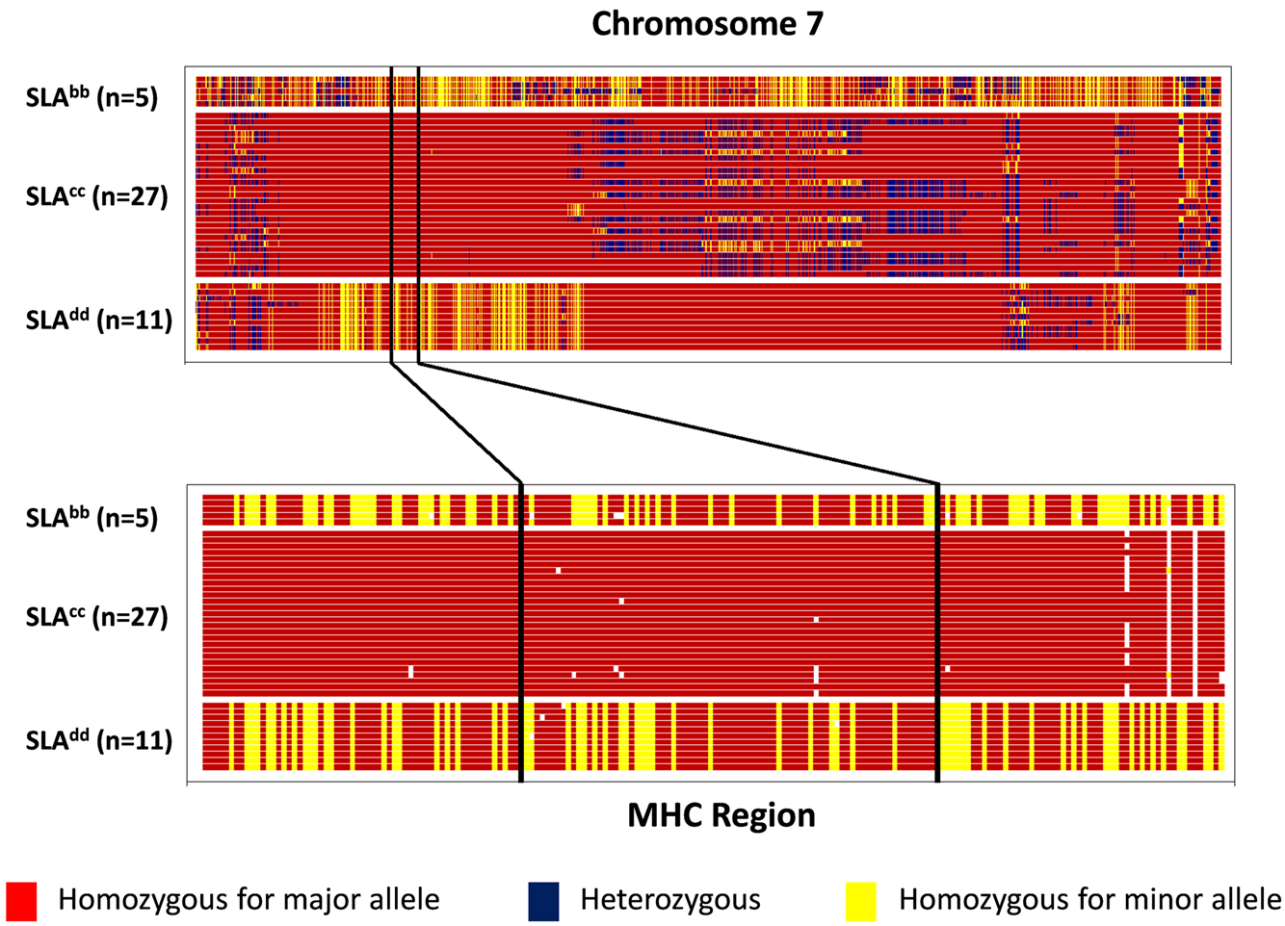
Genetic homozygosity map for chromosome 7 showing location of the MHC region in SLA^bb^, SLA^cc^ and SLA^dd^ line pigs. Each horizontal line represents a genotyped pig. Pigs lines are grouped in blocks. Vertical lines depict SNPs, colour-coded according to genotype. White lines represent ungenotyped SNPs or null alleles. A ‘major’ allele is the more frequent allele and a ‘minor’ allele is the less frequent allele of a pair of alleles within a given line. Locations of SNPs within the chromosome run in ascending numerical order from left to right.

A total of 5 SLA^bb^, 27 SLA^cc^, and 11 SLA^dd^ were genotyped. These pigs included donors, recipients and additional available non-transplanted pigs to increase the pool of samples for the homozygosity analysis. A total of 59,852 SNPs were successfully genotyped. The proportions of SNPs fixed (i.e. invariant) within each line were 0.86, 0.71 and 0.78 for the SLA^bb^, SLA^cc^ and SLA^dd^ lines respectively (Table 1). These were higher than the proportion fixed across all three lines (0.40), indicating that there has been independent fixation to different haplotypes within each line. The homozygosity map of chromosome 7 (Fig 1) confirms that lines are fully inbred across the region of the MHC, each for a different haplotype. Data for all autosomal chromosomes are shown in S1 Fig. The proportion of SNPs fixed within each chromosome varied from 0.65 (chromosomes 9 and 10 of the SLA^cc^ line) to 0.98 (chromosomes 13 and 16 of the SLA^bb^ line) (Table 1). The mean minor allele frequency (MAF) within chromosomes ranged from 0.10 to 0.39, a low mean MAF (e.g. 0.10 for chromosome 14 of the SLA^bb^ line) suggests that a chromosome is close to full homozygosity.

**Table 1:** Heterozygosity of SNPs on individual chromosomes of SLA^bb^, SLA^cc^ and SLA^dd^ lines of pig.

		Babraham SLA^bb^ (n = 5)			NIH minipig SLA^cc^ (n = 27)			NIH minipig SLA^dd^ (n = 11)		
Chromosome	No. of SNPs	Fixed^a^	Mean MAF^b^	Heterozygosity^c^	Fixed	Mean MAF	Heterozygosity	Fixed	Mean MAF	Heterozygosity
**1**	**6792**	**0.87**	**0.28**	**0.45**	**0.68**	**0.28**	**0.45**	**0.73**	**0.22**	**0.36**
**2**	**3179**	**0.72**	**0.32**	**0.37**	**0.77**	**0.26**	**0.40**	**0.93**	**0.12**	**0.24**
**3**	**2694**	**0.93**	**0.22**	**0.33**	**0.75**	**0.22**	**0.33**	**0.85**	**0.14**	**0.21**
**4**	**3645**	**0.90**	**0.39**	**0.38**	**0.70**	**0.24**	**0.35**	**0.79**	**0.35**	**0.55**
**5**	**2352**	**0.86**	**0.23**	**0.44**	**0.72**	**0.18**	**0.25**	**0.78**	**0.25**	**0.43**
**6**	**2849**	**0.75**	**0.34**	**0.51**	**0.66**	**0.20**	**0.30**	**0.72**	**0.32**	**0.53**
**7**	**3427**	**0.80**	**0.26**	**0.44**	**0.75**	**0.26**	**0.40**	**0.91**	**0.20**	**0.34**
**8**	**2554**	**0.78**	**0.38**	**0.60**	**0.80**	**0.25**	**0.39**	**0.81**	**0.27**	**0.40**
**9**	**3088**	**0.95**	**0.22**	**0.43**	**0.65**	**0.28**	**0.43**	**0.71**	**0.27**	**0.32**
**10**	**1571**	**0.87**	**0.21**	**0.42**	**0.65**	**0.29**	**0.38**	**0.78**	**0.21**	**0.33**
**11**	**1860**	**0.83**	**0.35**	**0.55**	**0.76**	**0.14**	**0.25**	**0.87**	**0.28**	**0.45**
**12**	**1475**	**0.80**	**0.22**	**0.41**	**0.77**	**0.33**	**0.50**	**0.79**	**0.27**	**0.37**
**13**	**3523**	**0.98**	**0.33**	**0.67**	**0.70**	**0.19**	**0.24**	**0.91**	**0.30**	**0.38**
**14**	**3947**	**0.92**	**0.10**	**0.21**	**0.70**	**0.24**	**0.33**	**0.72**	**0.24**	**0.44**
**15**	**2774**	**0.82**	**0.33**	**0.49**	**0.67**	**0.22**	**0.32**	**0.72**	**0.23**	**0.31**
**16**	**1816**	**0.98**	**0.14**	**0.29**	**0.67**	**0.21**	**0.35**	**0.78**	**0.31**	**0.54**
**17**	**1663**	**0.81**	**0.34**	**0.37**	**0.68**	**0.32**	**0.44**	**0.72**	**0.37**	**0.45**
**18**	**1284**	**0.74**	**0.38**	**0.64**	**0.79**	**0.33**	**0.45**	**0.88**	**0.26**	**0.42**
**X**	**1420**	**0.91**	**0.26**	**0.33**	**0.81**	**0.18**	**0.16**	**0.91**	**0.24**	**0.29**
**Unknown position**	**7939**	**0.84**	**0.30**	**0.46**	**0.70**	**0.24**	**0.34**	**0.79**	**0.25**	**0.39**
**Total**	**59852**	**0.86**^d^	**0.28**	**0.44**	**0.71**	**0.24**	**0.35**	**0.80**	**0.26**	**0.39**

^a^ Proportion of SNPs homozygous within the line

^b^ Minor allele frequency for SNPs still segregating within the line

^c^ Proportion of pigs heterozygous for a given SNP still segregating within the line

^d^ Overall value across the genome

### Association of SNP matching status with rejection

**Fig 2:**
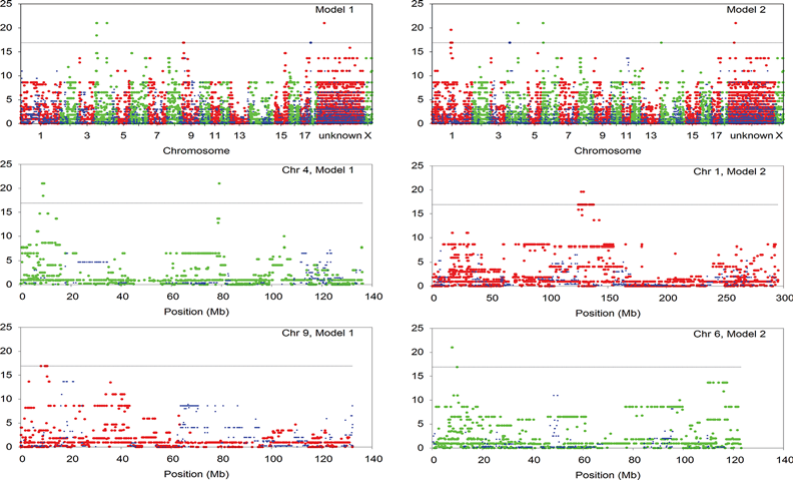
Manhattan plots showing the LRT values from the test of association of SNP mismatches with outcome of corneal transplantation assuming rejection Models 1 and 2. The two upper plots show values for the entire genome except the Y chromosome. For SNP mismatches associated with rejection, LRT values of adjacent chromosomes are distinguished by alternating red and green symbols. Blue symbols on all plots represent values for SNP mismatches associated with acceptance. Lower plots represent individual chromosomes in which there were blocks of 4 or more SNP mismatches associated with rejection. The horizontal line on each plot indicates the genome-wide significance LRT threshold obtained using permutation analysis (16.9).

We previously reported that in the SLA^bb^ to SLA^cc^, SLA^dd^ to SLA^cc^ and SLA^cc^ to SLA^cc^ line combinations respectively, 4/6, 6/10 and 0/5 grafts progressed to immunological rejection [10]. Twenty of 21 recipients were included in the GWA analysis, one being excluded because extracted DNA was of inadequate quality for allo-typing. The segregation of approximately 60% of the SNPs across the three lines allowed good coverage of the whole genome for the GWA analysis, the numbers of SNPs used being 35212 and 36036 when assuming model 1 and model 2 respectively. The genome-wide 5% significance LRT threshold calculated with 100,000 permutations was 16.9 for both models, as indicated in Manhattan plots of the SNP distribution (Fig 2).

Sixty one SNPs were significantly associated with corneal graft outcome (Table 2). For rejection Model 1, mismatches significantly associated with rejection (SNP_rej_) occurred in blocks on chromosome 4 (7 SNPs; 6 spanning 0.3 megabase pairs (Mbp) and a further SNP 7Mbp downstream from these) and chromosome 9 (7 SNPs spanning 2.4Mbp). For rejection Model 2, SNP_rej_ were located on chromosomes 1 (37 SNPs spanning 13.4Mbp and approximately 115 known genes), 4 (1 SNP, also significant in model 1), 6 (4 SNPs spanning 2.1Mbp) and 14 (1 SNP). The SNP_rej_ in each closely linked block of SNPs were occasionally adjacent, but more frequently interspersed with SNPs that had non-significant LRT scores (Fig 2). Interestingly, there were no SNP_rej_ on chromosome 7, the location of the MHC, in either model (Fig 2 and Table 2). Chromosomal locations were unknown for 1 SNP_rej_, which appeared in both models (Table 2). There were also 3 SNPs within 0.5Mb of each other on chromosome 4 (Model 2) and 1 SNP on chromosome 18 (Model 1) in which a mismatch was significantly associated with graft acceptance (SNP_acc_). LRT values for the full genome scan in Models 1 and 2 are shown in S2 Fig and S3 Fig respectively; SNPs significantly associated with rejection are highlighted. The permutation analysis to determine significance threshold is also shown for each model.

**Table 2:** SNP loci at which mismatches between donor and recipient are significantly associated with rejection (57 SNPs) or acceptance (4 SNPs) of a corneal graft.

Chromosome	SNP location^a^	Significant in	
		Model 1	Model 2
**1**	**132826911**		**√**
**1**	**133258994**		**√**
**1**	**133099036**		**√**
**1**	**133084921**		**√**
**1**	**133326538**		**√**
**1**	**133472365**		**√**
**1**	**134378501**		**√**
**1**	**134796719**		**√**
**1**	**135375075**		**√**
**1**	**0**		**√**
**1**	**0**		**√**
**1**	**135569814**		**√**
**1**	**135930910/136076989**		**√**
**1**	**136240599**		**√**
**1**	**136427159**		**√**
**1**	**136639460**		**√**
**1**	**136780600**		**√**
**1**	**137134551**		**√**
**1**	**137512402**		**√**
**1**	**137887750**		**√**
**1**	**138686941**		**√**
**1**	**139295346**		**√**
**1**	**140022043/140141190**		**√**
**1**	**140052792/140172769**		**√**
**1**	**0**		**√**
**7**^b^	**29490937**		**√**
**1**	**142944521**		**√**
**1**	**142970565**		**√**
**1**	**143601266**		**√**
**1**	**144487578**		**√**
**1**	**144865731**		**√**
**1**	**144888036**		**√**
**1**	**145285984**		**√**
**1**	**145605557**		**√**
**1**	**145711971**		**√**
**1**	**137462211**		**√**
**1**	**146247253**		**√**
**4**	**9649533**	**√**	
**4**	**9885672**	**√**	
**4**	**10283814**	**√**	
**4**	**10331873**	**√**	
**4**	**10355258**	**√**	
**4**	**10423196**	**√**	
**4**	**83279186**	**√**	**√**
**6**	**10893273**		**√**
**6**	**10937617**		**√**
**6**	**11003757**		**√**
**6**	**13664912**		**√**
**9**	**9183235**	**√**	
**9**	**11133568**	**√**	
**9**	**11355126**	**√**	
**9**	**11394193**	**√**	
**9**	**0**	**√**	
**9**	**11670612**	**√**	
**9**	**11819832**	**√**	
**14**	**1404225**		**√**
**0**^c^	**0**	**√**	**√**
**4**^d^	**19490147**		**√**
**4**^d^	**19517211**		**√**
**4**^d^	**19917508**		**√**
**18**^d^	**11099460**	**√**	

^a^ Locations derived from Sscrofa 10.2, Ensembl Release 83.

^b^ SNP mapped to chromosome 1 in Sscrofa 9 assembly

^c^ Chromosomal location unknown

^d^ SNPs associated with acceptance of a graft

Further examination of the data showed that within each genomic block of closely linked SNP_rej_ the distribution of donor-recipient pairs between the four outcome groups (matched-accepted, matched-rejected, mismatched-accepted, mismatched-rejected) was identical, but that there was variation between genomic blocks (S1 Table). In all genomic blocks, at least 18/20 donor-recipient pairs were in either matched-accepted or mismatched-rejected groups, indicating that typing pigs for any of these three blocks of SNPs before transplantation would permit prediction of transplant success or failure with 90% accuracy. The remaining one or two pairs of pigs in each block were in the mismatched-accepted group (i.e. there remained the possibility of failure had grafts been monitored for longer than the experimental cut-off point of 90 days), except in one genomic block (chromosome 4, model 1) where there was one pair in the matched-rejected group. Donors and recipients in the accepted SLA^cc^ to SLA^cc^ graft group were matched for all polymorphisms significantly associated with rejection.

### Conservation of synteny between pig, human and mouse genomes

**Fig 3:**
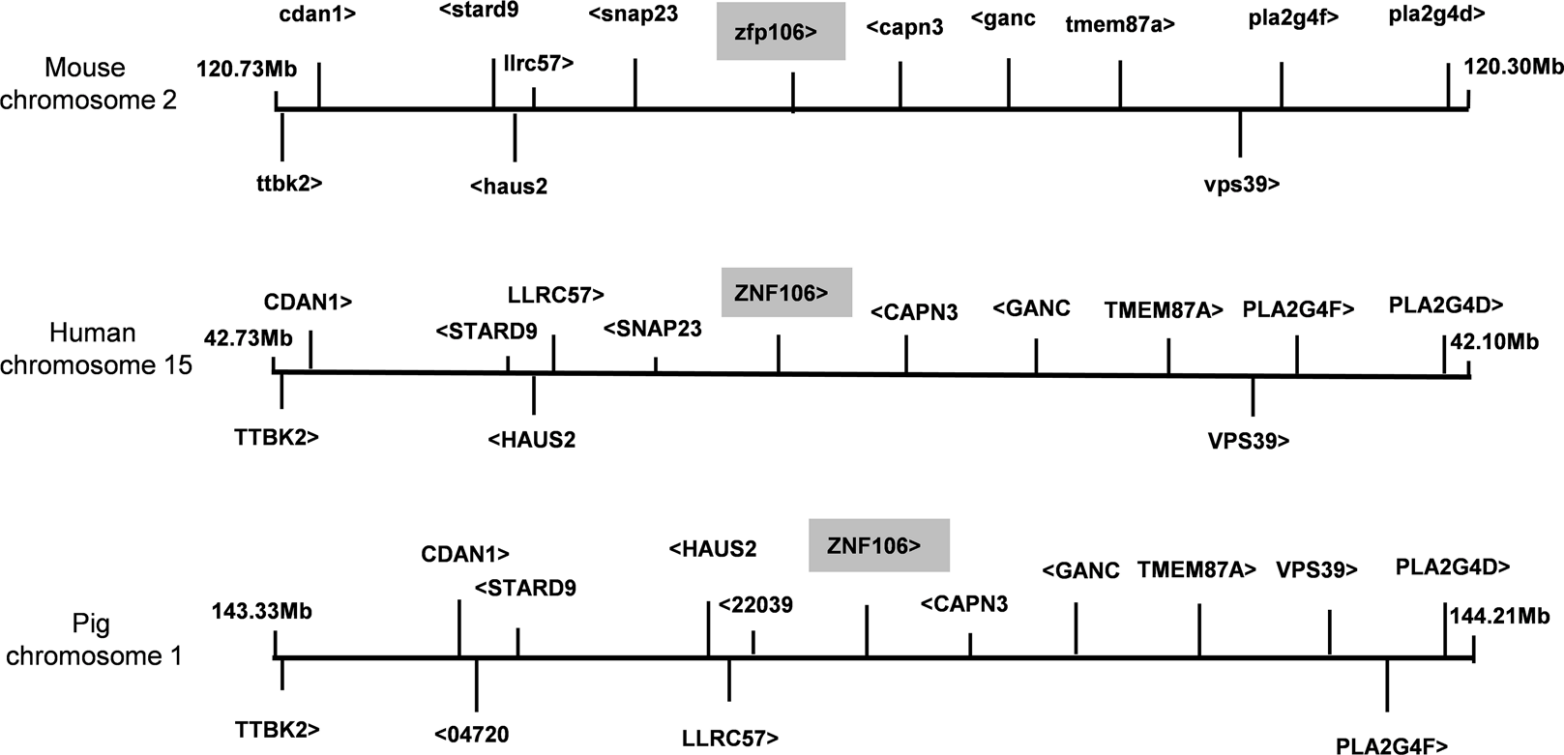
Conservation of synteny of genomes in the region of the *ZNF106* (*Zfp106*) gene (Ensembl 83; www.ensembl.org). Only protein coding genes are shown. The protein 04720 in the pig genome is uncharacterised, while 22039 bears homology to human SNAP23 but is truncated. Arrowheads indicate direction of transcription.

The pig genome is still not fully annotated. However, the major blocks of SNP_rej,_ on chromosomes 1, 4, and 9, showed clear conservation of synteny with regions of human chromosomes 15, 8 and 11 and mouse chromosomes 2, 15 and 7 respectively (Ensembl 83; www.ensembl.org). Regions on pig chromosomes 1 and 4 and the homologous regions in the human and mouse genomes have a particularly high density of genes. This is exemplified by Fig 3 which shows a 1Mbp span either side of the pig *ZNF106* gene, located 2.4Mb from the 3’ end of the 13.4Mbp block of SNP_rej_ on chromosome 1, together with homologous human and mouse regions. The mouse homologue of *ZNF106*, *Zfp106*, codes for H-3a the minor antigen associated with corneal graft rejection in mice [9]. Indeed, this block of SNP_rej_ also includes the beta-2-microglobulin (*β2-M*) gene, the mouse homologue of which also contains allograft-defined polymorphisms [17].

## Discussion

GWA analysis has revealed at least four independently segregating non-MHC regions of the pig genome containing genetic polymorphisms associated with corneal graft rejection, three of which have not been previously identified. Two of the four regions (Chr 1, Model 2 and Chr 9, Model 1) bear close homology to regions in humans and mice containing a high density of protein coding genes. One of these (Chr 1) containing approximately 115 genes, includes homologues of genes in the H-3 region of the mouse which have already been associated with corneal graft rejection (*Zfp106 [9]*) and skin graft rejection (*β-2M [18]* respectively). Chr 9:9183235–11819832) contains at least 22 protein coding genes with close homology to human and mouse genes on chromosomes 11 and 7 respectively, none of which have been associated with transplant rejection in any species. The current Sus scrofa genome build (10.2) defines SNPs in the remaining two regions (Chr 4 and Chr 6) as intergenic.

The outcome is consistent with rodent data implicating MHags as major contributors to corneal graft rejection [5, 6], with estimates of involvement of 3 or 4 immunodominant loci [19]. It is also consistent with the overall failure of human MHC matching studies to show clinical benefit and further validates the use of GWAS as a tool for identifying Mhags. In swine, minor antigens have also been implicated in renal [20] and skin graft rejection [21].

The data revealed a relatively high level of homozygosity within each line of pig, although the reliability of homozygosity measurement of the SLA^bb^ Babraham line, in particular, was constrained by the relatively small numbers of pigs available. Despite the high level of homozygosity observed, there was considerable inter-line variability and these factors together explain how there was sufficient power to identify these loci with a relatively small number of animals. The success of the five MHC-matched intra-line SLA^cc^ to SLA^cc^ grafts is explained by the finding that donors and recipients of these grafts were all matched at the minor loci identified.

A previous study by DNA profiling has shown the Babraham line to be relatively homozygous [12], inbreeding having commenced in the UK in the 1970s. However, there are no published genetic homozygosity data pertaining to the two NIH minipig lines, SLA^cc^ and SLA^dd^, the founders of which originated in the USA from MHC-disparate lines derived from a cross between two unrelated, outbred lines [11]. Once the MHC was fixed within lines, they were randomly bred to maintain genetic heterozygosity [22]. The UK herds were established from limited breeding nuclei of 2 boars and 4 gilts of each line imported from the USA in 1992. Thus, the non-MHC heterozygosity within each line may be diminished compared with the parental lines as a consequence of a genetic ‘bottleneck’.

The minor locus SNP_rej_ regions identified, mostly in blocks ranging in size from 2–13 Mbp, presumably contain genes associated with adaptive immune responses to ‘non-self’, either by coding for MHags directly and illiciting T cell-mediated immunity (Model 1 hypothesis) or by altering the activity of genes that control immune responses (Model 2 hypothesis). The fact that there were few SNPs common to both models is not surprising since in Model 2 no account was taken of the direction of mismatch. Thus considerably more donor-recipient pairs would be considered mismatched in this category, resulting almost always in a different distribution of donor-recipient pairs in the four outcome groups for a given SNP.

Based on our current understanding of the mechanisms of rejection, we can offer no immunological explanation for the association of 4 SNP mismatches with graft acceptance. As the SNP chip only assays a small fraction of the >28 million putative SNPs currently annotated in the pig genome (http://www.ensembl.org/Sus_scrofa/Info/Annotation), all the SNPs identified are likely to be markers through linkage for one or more nearby causative loci. It has been shown in the context of both mouse and human graft rejection that MHags usually comprise both CD4 and CD8 epitopes that are not necessarily in the same gene [23]. Therefore, for each minor mismatch block there may be more than one causative locus. Indeed, it seems unlikely that a single minor locus gene would be responsible for the 13 Mbp block of SNP_rej_ on chromosome 1 (Model 2) homologous to the mouse H-3 region, (with linkage accounting for the remaining SNP_rej_), due to the block size (encompassing approximately 115 genes). The mouse H-3 region contains at least 3 minor loci: H-3a (a cytotoxic lymphocyte (CTL) epitope within Zfp106 *[8])*; H-3b (a CD4 epitope encoded by a gene close or identical to the *Pcsk2* gene [8, 24]); and *β2-M*. The pig SNP_rej_ encompass the equivalent of H3a and *β2-M* loci but not the *PCSK2* region, which lies on chromosome 17, and which in man also lies on a different chromosome from *ZNF106* and *β2-M*. Recent re-sequencing of multiple pig genomes has revealed a further 28 million putative SNPs, including 430 within the pig *ZNF106* locus (http://www.ensembl.org/Sus_scrofa/Gene/Variation_Gene/Table?db=core;g=ENSSSCG00000004725;r=1:143786677-143847965;t=ENSSSCT00000005218). The 430 putative SNPs in the pig *ZNF106* gene include 11 putative missense SNP variants. The extent to which graft rejection in different species is cause by polymorphisms in homologous genes is uncertain and identifying whether rejection in the pig could be accounted for by polymorphisms in the swine *ZNF106* and *β2-M* genes would require sequencing of these genes in our donors and recipients and functional studies. However, the close homology between the SNP_rej_ region on chromosome 1 and a well-documented minor histocompatibility region in the mouse containing Zfp106, previously implicated in mouse corneal graft rejection [9], and the conservation of synteny between pig, mouse and human genomes in this region, suggests that polymorphisms in the *ZNF106* gene and/or other genes in this region (such as *β2-M*) may contribute to corneal graft rejection in all three species.

Irrespective of whether critical polymorphisms are located in these genes in swine, it is noteworthy that SNP_rej_ on chromosome 1 were revealed only in the Model 2 analysis, which did not conform to a T cell epitope hypothesis. We thus anticipate that loci highlighted by SNP_rej_ in Model 2 may be immune response genes, such as micro RNAs, transcription factors or other immunoregulatory proteins, or ligands of NK receptors outside the MHC region. In the context of the Model 2 hypothesis, it is noteworthy that polymorphisms in mouse β2-M can modulate immune responses by influencing indirectly the effectiveness of peptide binding and therefore of both self and allogeneic peptide selection for presentation on MHC class I molecules [25, 26]. In addition, β2-M polymorphisms have the potential to modulate NK cell killing as well as T cell activation, with or without donor-recipient MHC incompatibility.

The evidence from this study justifies further testing of the model, whereby donors and recipients are typed before transplantation and outcome is predicted according to SNP disparity. For example, our results suggest that pre-transplant typing and selection for matches or mismatches between donor and recipient at any of the three largest blocks of SNPs, i.e. chromosomes 4 or 9 (Model 1) or chromosome 1 (Model 2) would permit prediction of graft survival or rejection with 90% accuracy (18/20 cases). Similarly, typing of donors and recipients and deliberate selection of SLA^cc^ to SLA^cc^ (or other intra-line) grafts to be mismatched at some or all of these loci would test whether this rejection on account of minor mismatches was truly independent of MHC disparity. Furthermore, we have previously shown that some allografts can contain significantly elevated numbers of T cells without showing clinical sign of rejection [10]. Thus, should the predictive capacity of the model be confirmed, it offers possibilities to investigate early systemic or local events that determine whether a graft will be rejected or accepted.

In summary, the study demonstrates for the first time the power of GWA to reveal independently segregating minor histocompatibility regions associated with corneal graft rejection, using a model that more closely resembles human genetic variability between donor and recipient than do fully inbred rodent models. The regions identified contained homologues of mouse genes associated with rejection, as well as genes that have not so far been associated with rejection in other species. The exact minor locus genes accounting for swine transplant outcome have yet to be identified, but we suggest that the level of precision we have achieved is sufficient to merit a comparable human study, focusing initially on regions of the genome that share homology with pig SNP_rej_ regions. This would determine whether there was true species overlap of genes controlling rejection and whether it would be of value to type and match at minor loci to improve outcome in human corneal transplantation.

## Supporting Information

S1 Fig Maps of homozygosity status for the three pigs lines SLA^bb^, SLA^cc^ and SLA^dd^. (PPTX)

S2 Fig LRT values for the full genome scan in Model 1. (XLSX)

S3 Fig LRT values for the full genome scan in Model 2. (XLSX)

S1 Table Distribution of donor-recipient pairs between the four outcome groups. (XLSX)
