# Supplementary material for: Genome-Wide Analysis in Swine Associates Corneal Graft Rejection with Donor-Recipient Mismatches in Three Novel Histocompatibility Regions and One Locus Homologous to the Mouse H-3 Locus
Source: PLoS One. 2016 Mar 24;11(3):e0152155. doi: 10.1371/journal.pone.0152155 (PMC4806994; doi:10.1371/journal.pone.0152155)
Supplement: S1 Fig — (PPTX) [file pone.0152155.s001.pptx]

## Slide 1
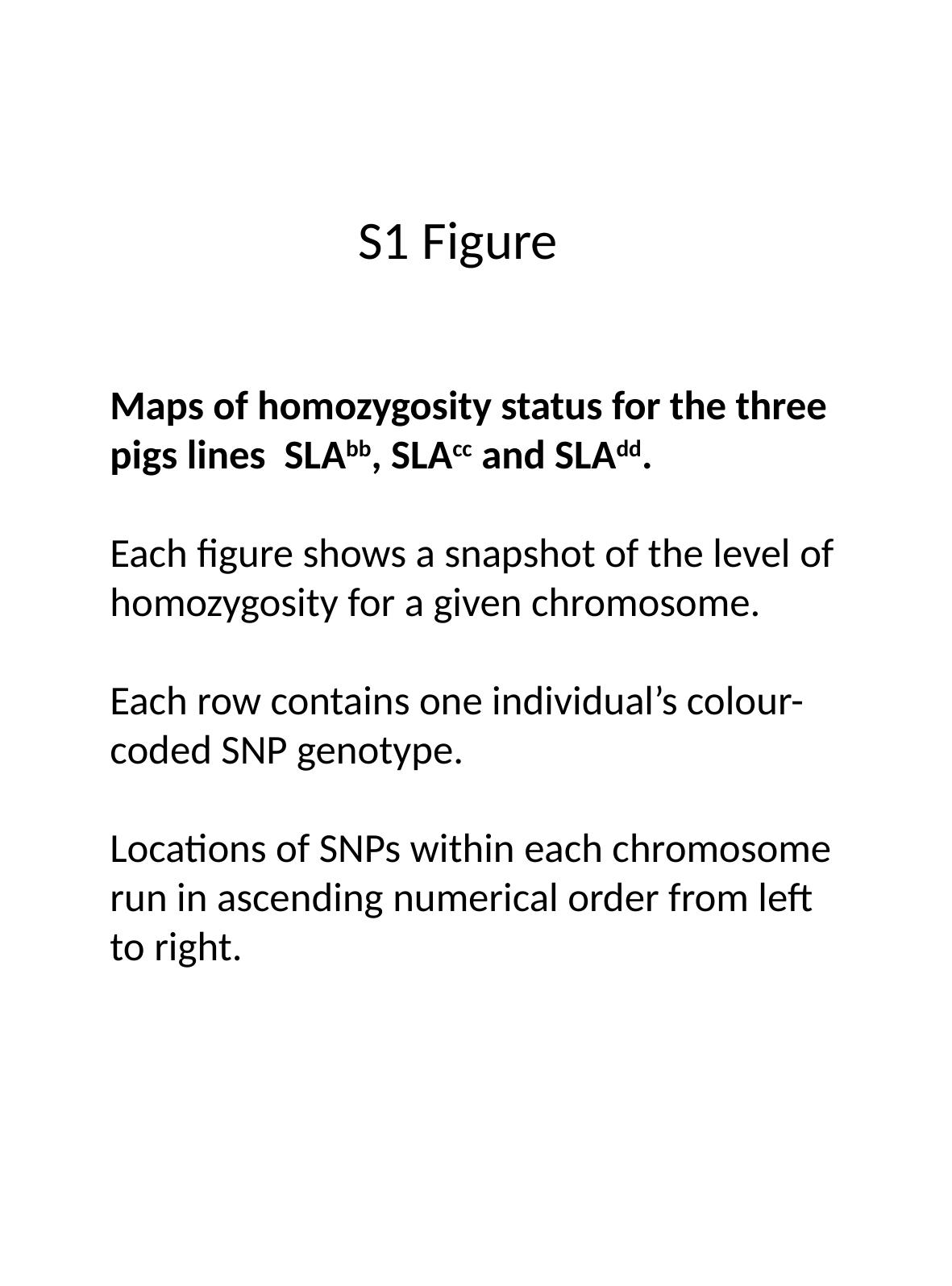

S1 Figure
# Maps of homozygosity status for the three pigs lines SLAbb, SLAcc and SLAdd. Each figure shows a snapshot of the level of homozygosity for a given chromosome. Each row contains one individual’s colour-coded SNP genotype. Locations of SNPs within each chromosome run in ascending numerical order from left to right.

## Slide 2
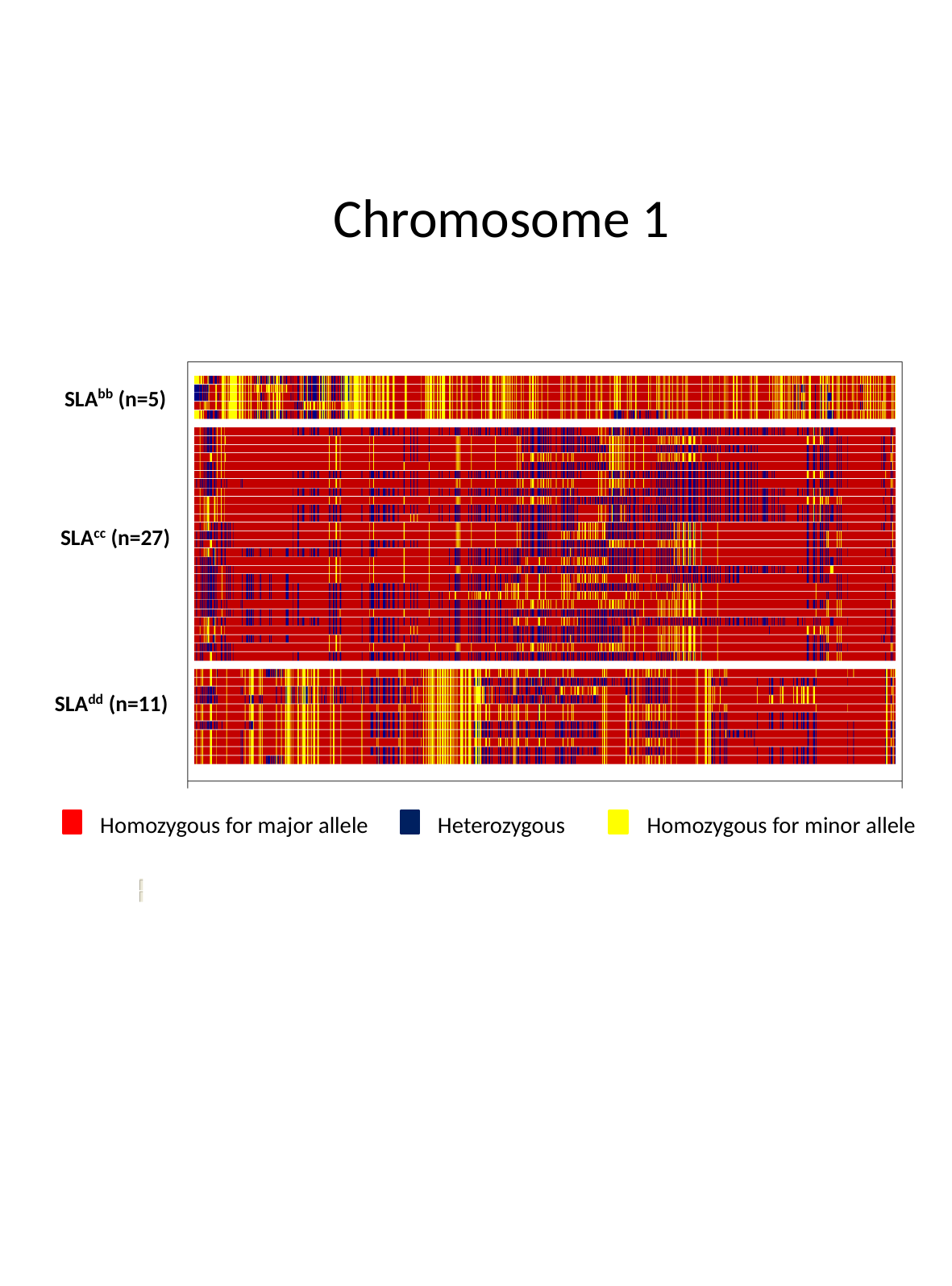

# Chromosome 1
SLAbb (n=5)
SLAcc (n=27)
SLAdd (n=11)
Homozygous for major allele
Heterozygous
Homozygous for minor allele

## Slide 3
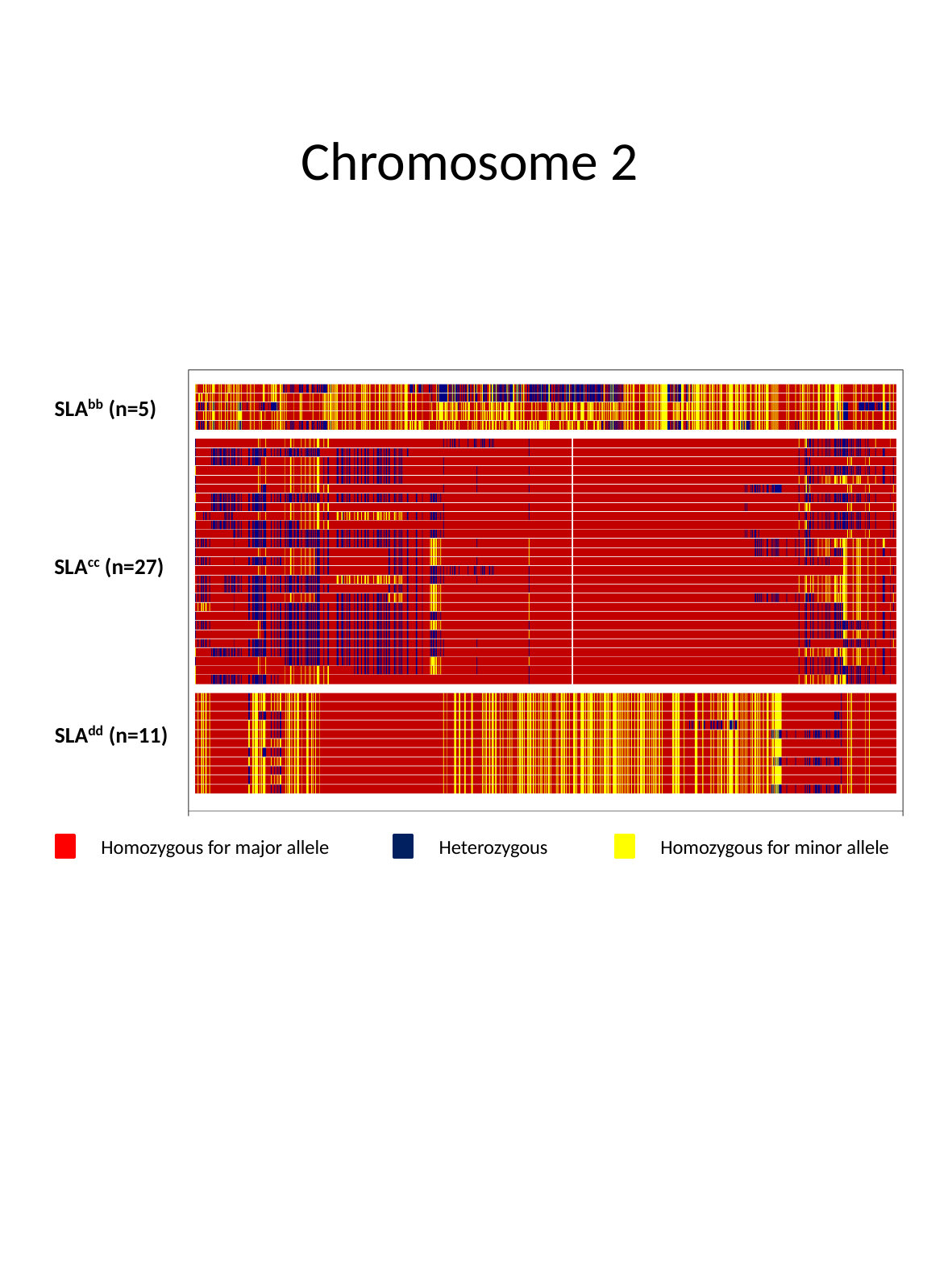

# Chromosome 2
SLAbb (n=5)
SLAcc (n=27)
SLAdd (n=11)
Homozygous for major allele
Heterozygous
Homozygous for minor allele

## Slide 4
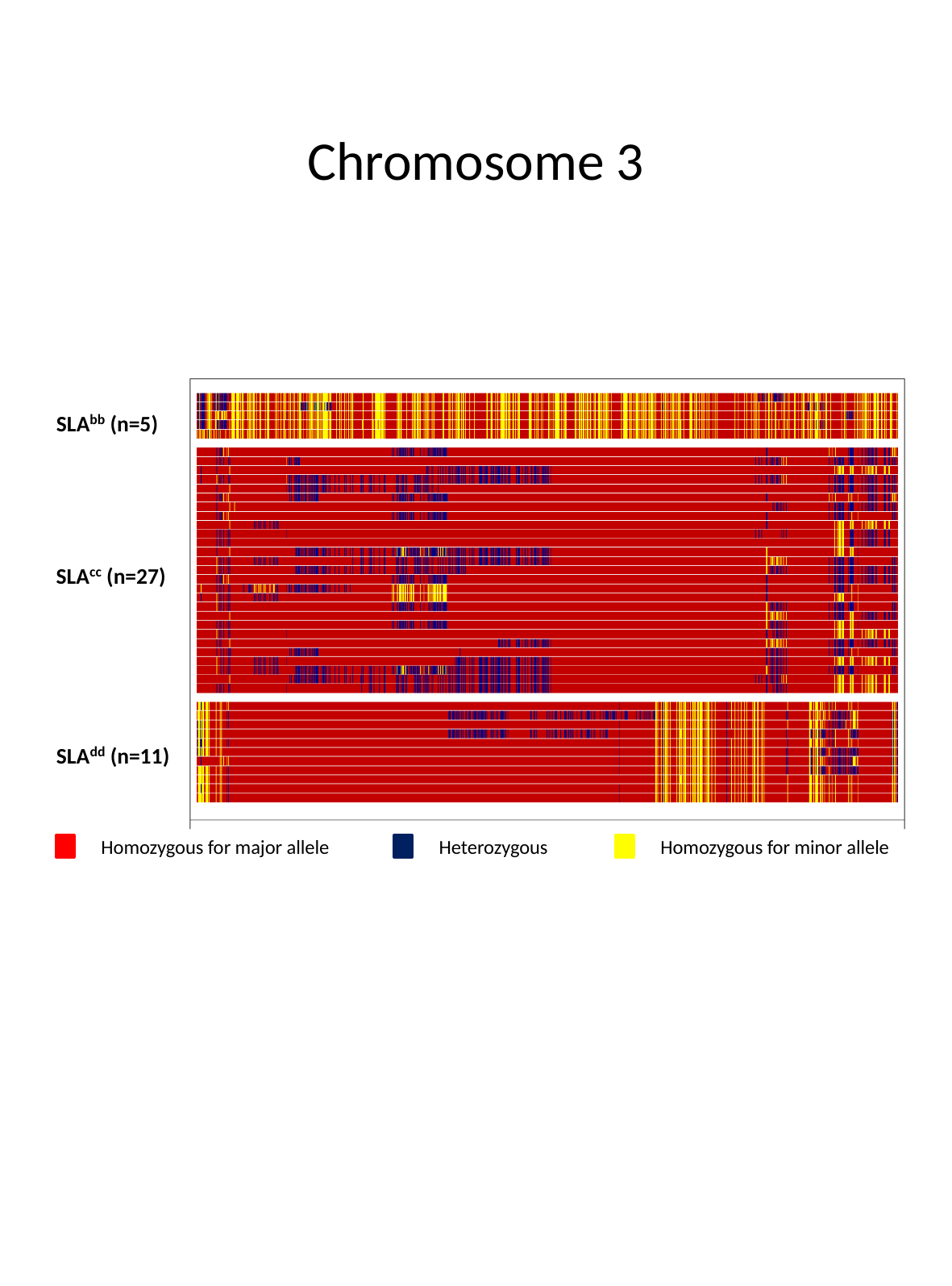

# Chromosome 3
SLAbb (n=5)
SLAcc (n=27)
SLAdd (n=11)
Homozygous for major allele
Heterozygous
Homozygous for minor allele

## Slide 5
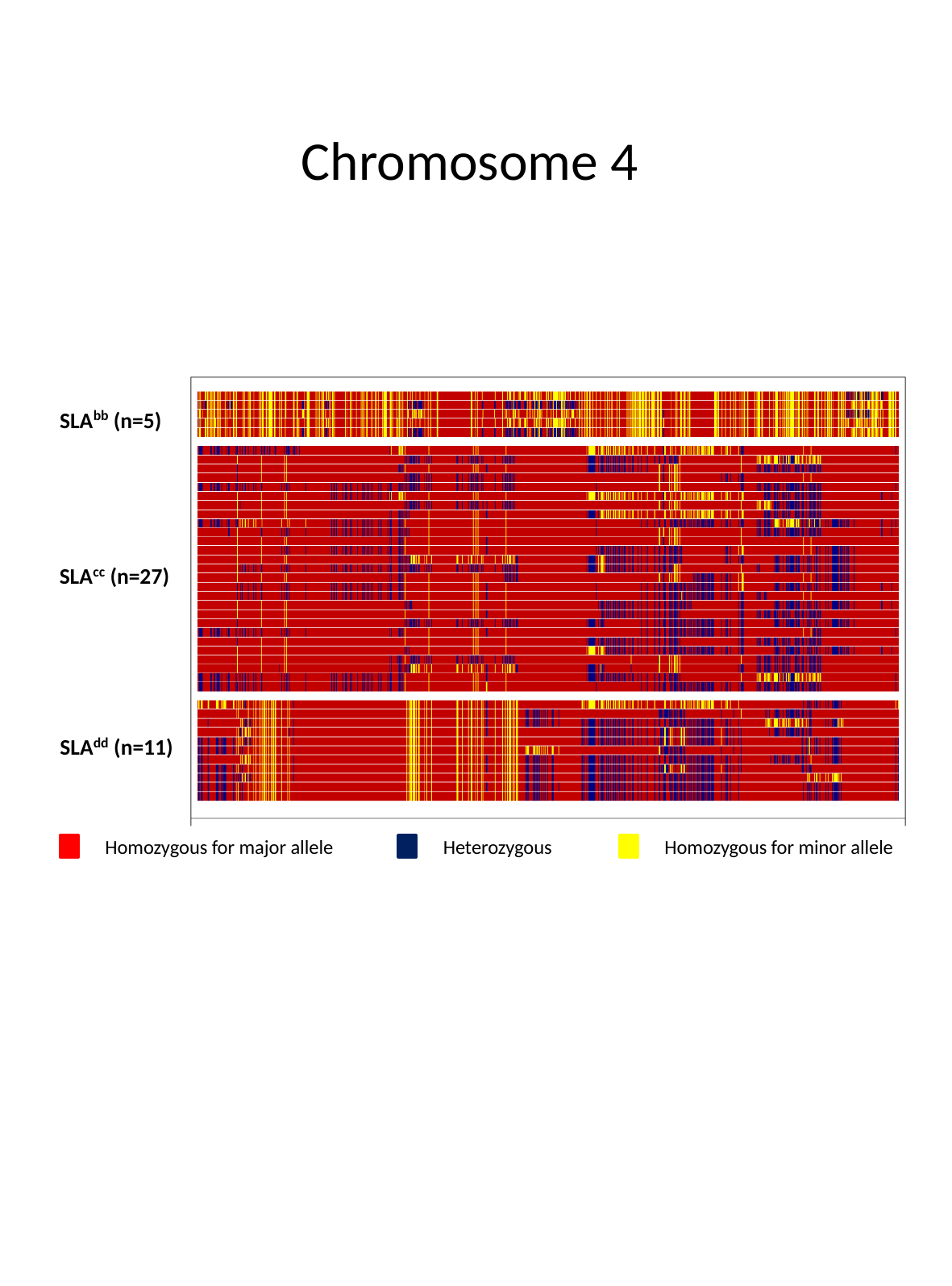

# Chromosome 4
SLAbb (n=5)
SLAcc (n=27)
SLAdd (n=11)
Homozygous for major allele
Heterozygous
Homozygous for minor allele

## Slide 6
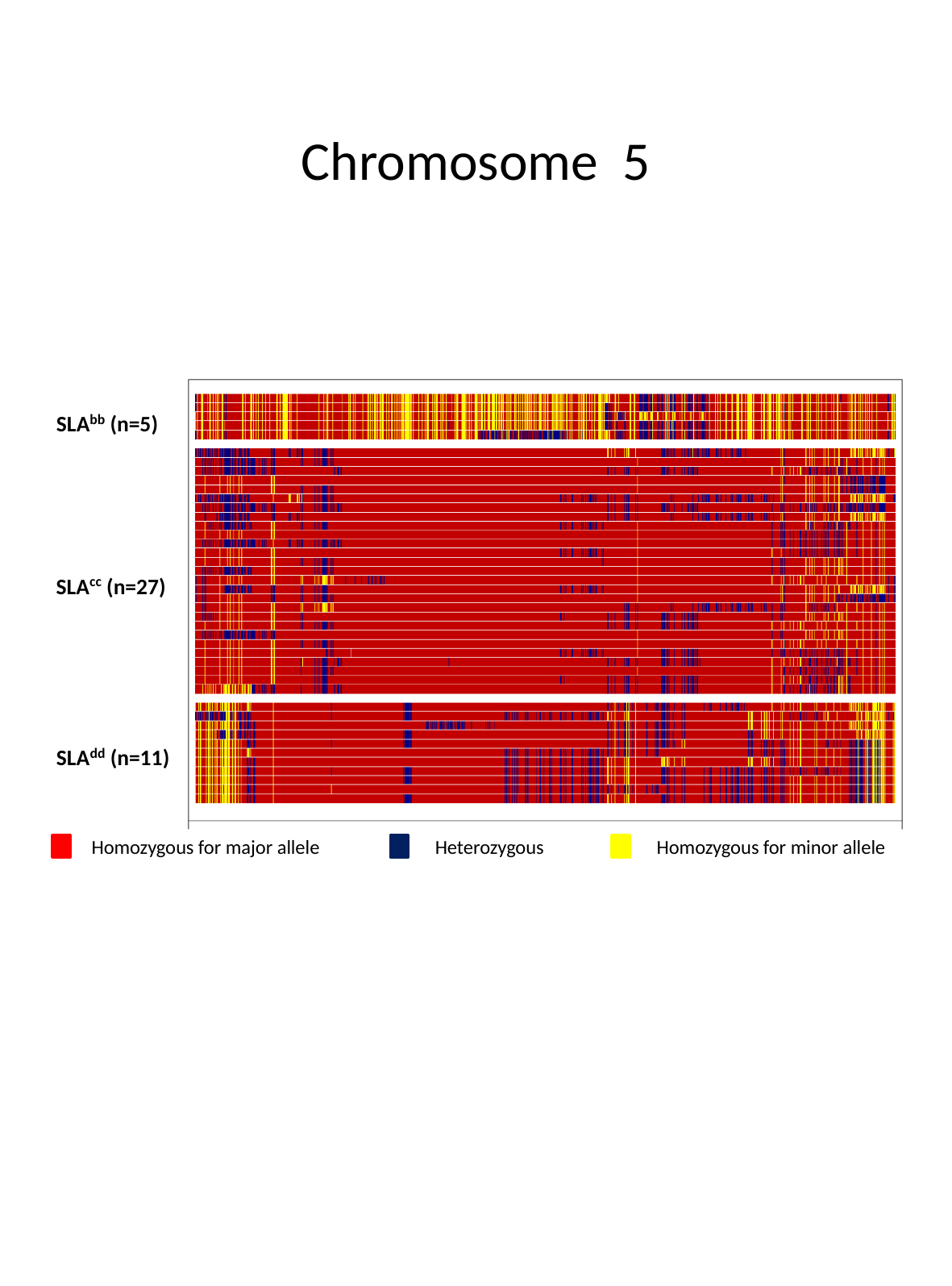

# Chromosome 5
SLAbb (n=5)
SLAcc (n=27)
SLAdd (n=11)
Homozygous for major allele
Heterozygous
Homozygous for minor allele

## Slide 7
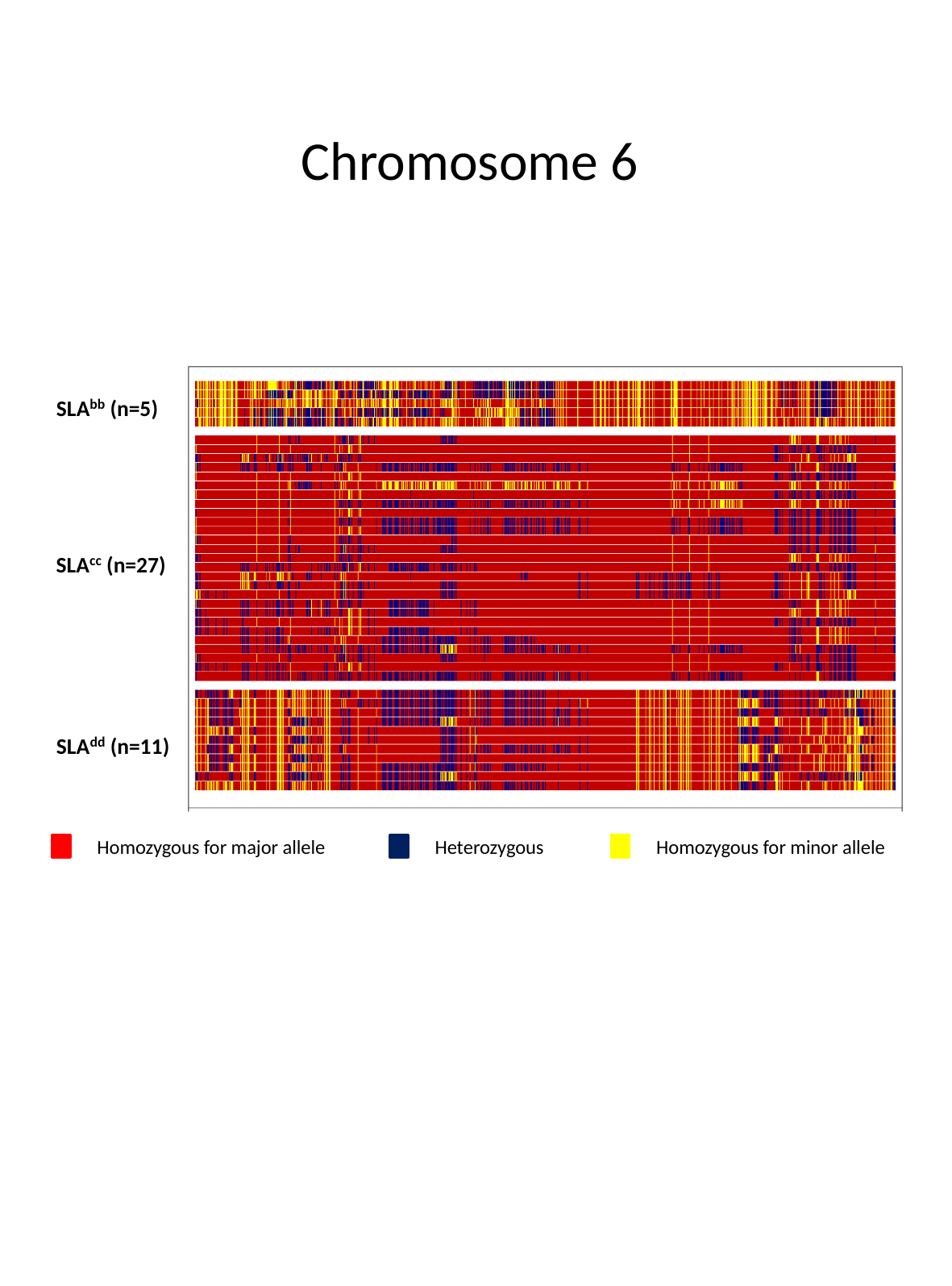

# Chromosome 6
SLAbb (n=5)
SLAcc (n=27)
SLAdd (n=11)
Homozygous for major allele
Heterozygous
Homozygous for minor allele

## Slide 8
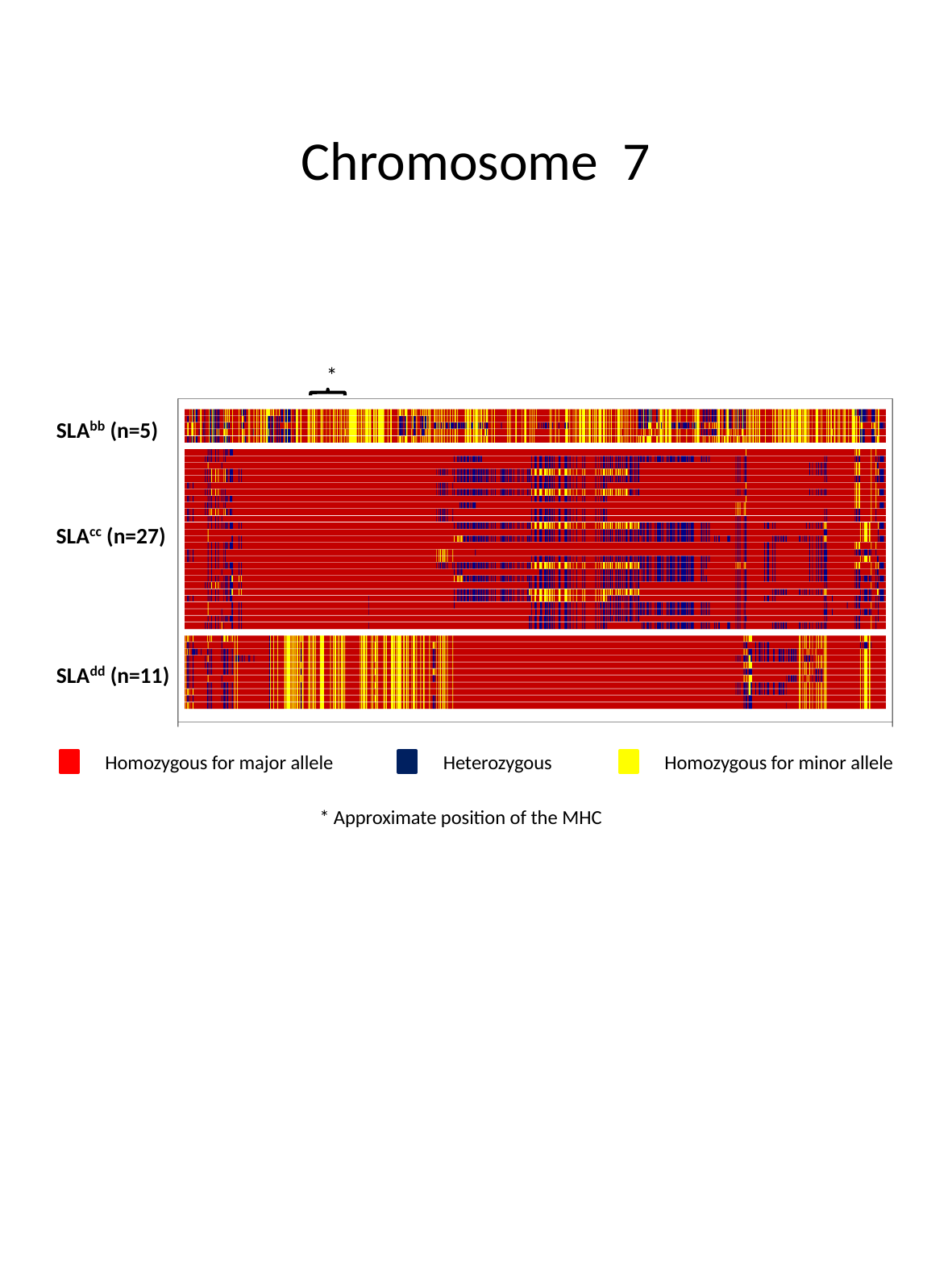

# Chromosome 7
*
SLAbb (n=5)
SLAcc (n=27)
SLAdd (n=11)
Homozygous for major allele
Heterozygous
Homozygous for minor allele
* Approximate position of the MHC

## Slide 9
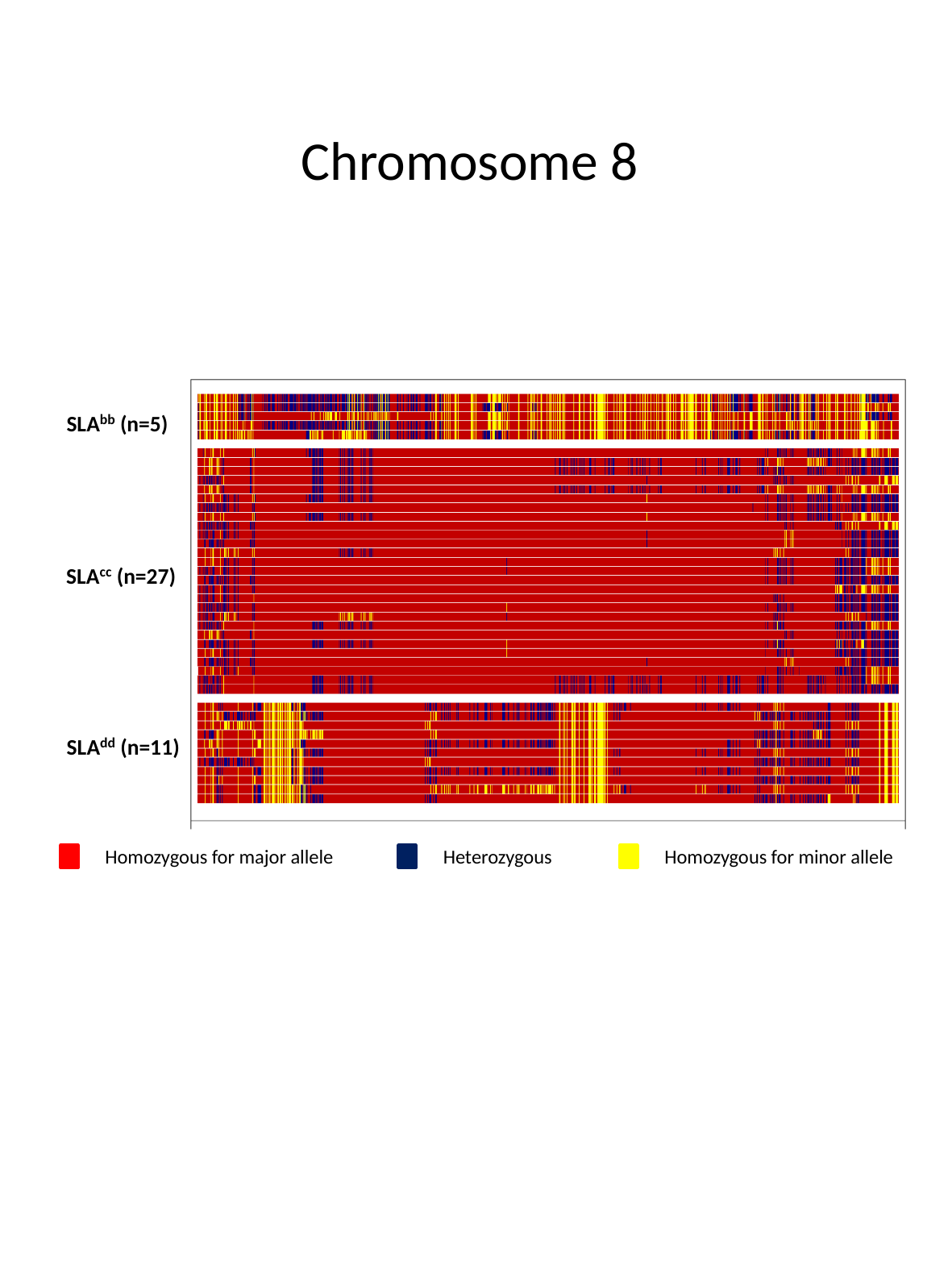

# Chromosome 8
SLAbb (n=5)
SLAcc (n=27)
SLAdd (n=11)
Homozygous for major allele
Heterozygous
Homozygous for minor allele

## Slide 10
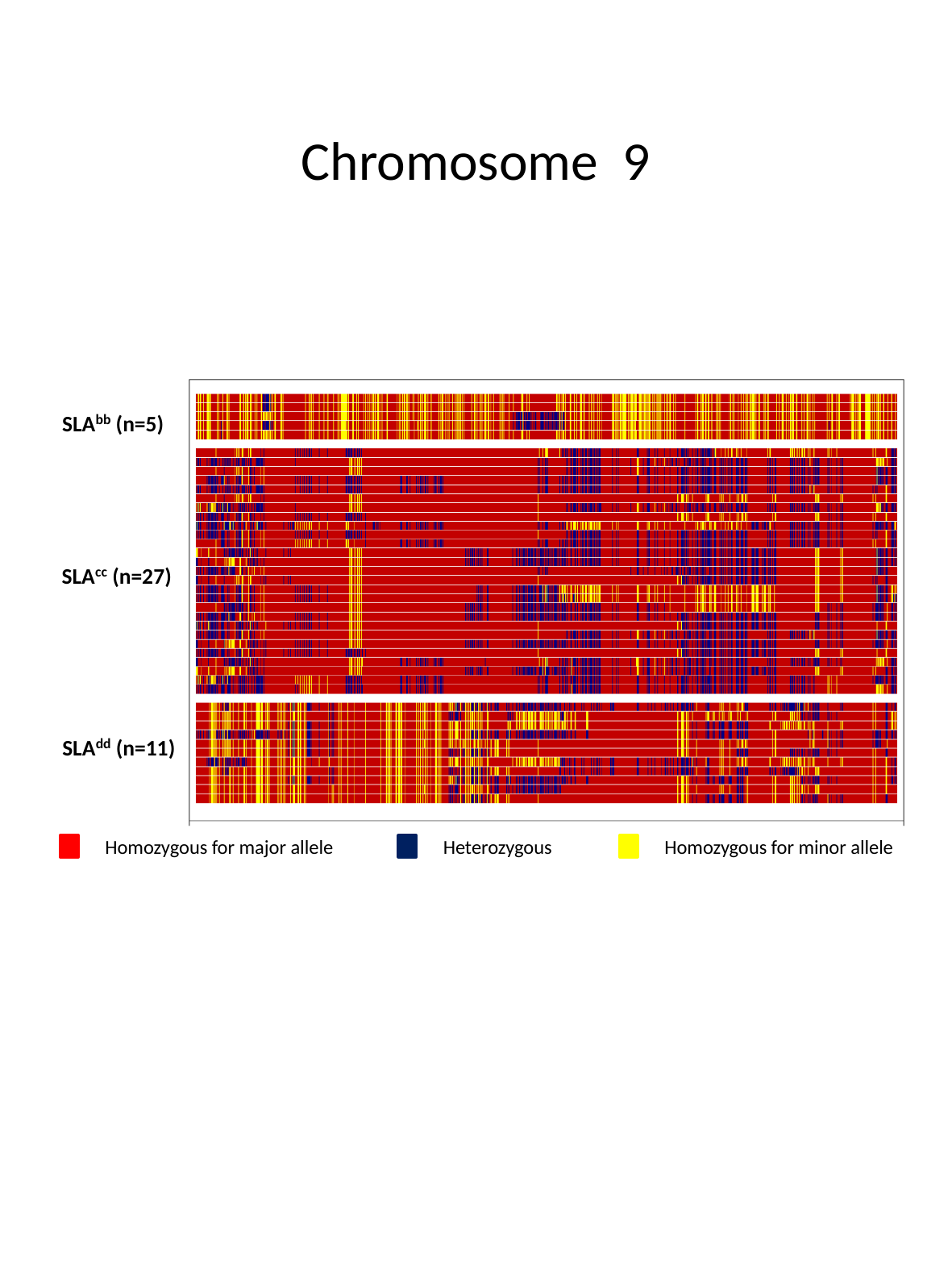

# Chromosome 9
SLAbb (n=5)
SLAcc (n=27)
SLAdd (n=11)
Homozygous for major allele
Heterozygous
Homozygous for minor allele

## Slide 11
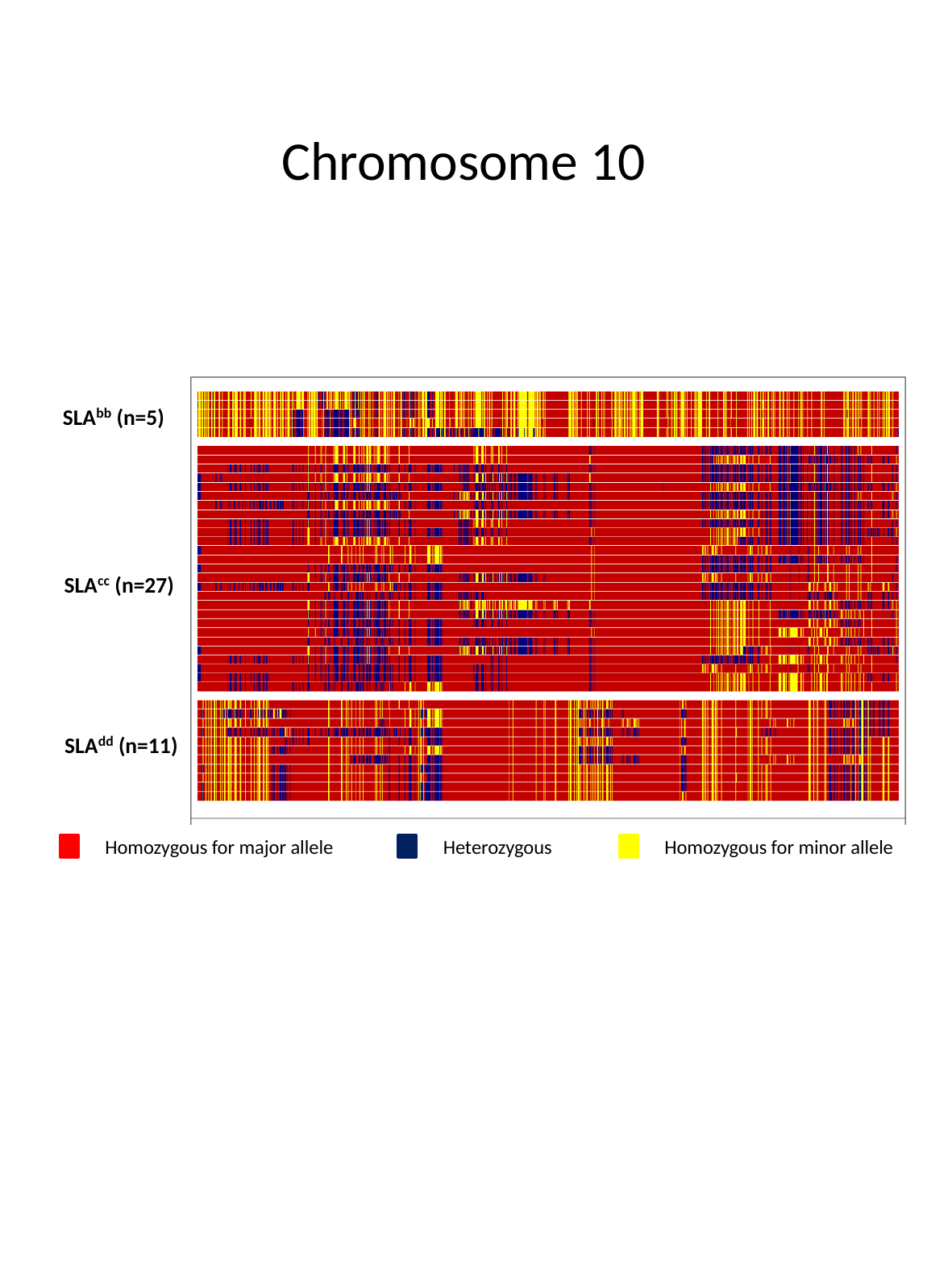

# Chromosome 10
SLAbb (n=5)
SLAcc (n=27)
SLAdd (n=11)
Homozygous for major allele
Heterozygous
Homozygous for minor allele

## Slide 12
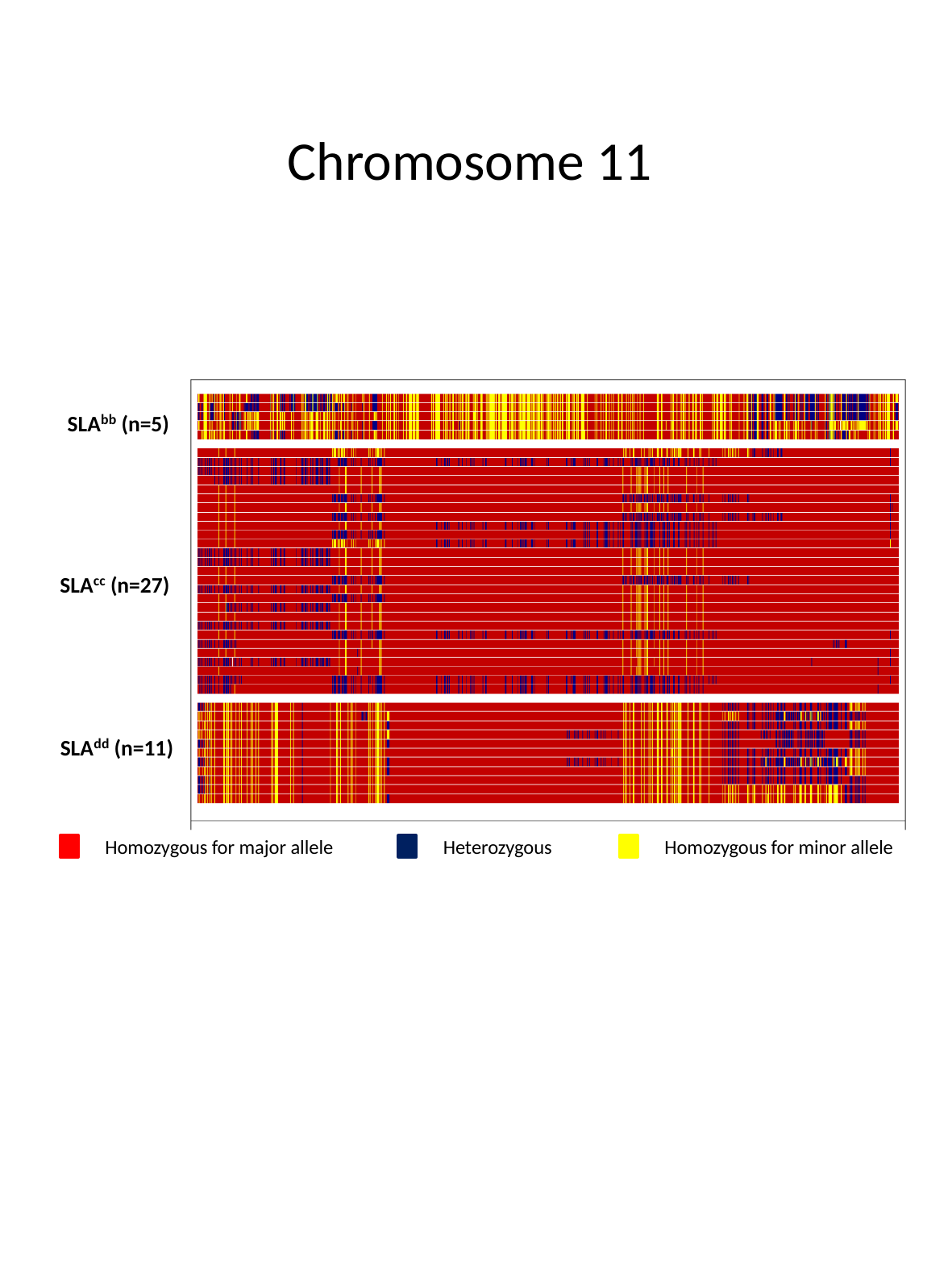

# Chromosome 11
SLAbb (n=5)
SLAcc (n=27)
SLAdd (n=11)
Homozygous for major allele
Heterozygous
Homozygous for minor allele

## Slide 13
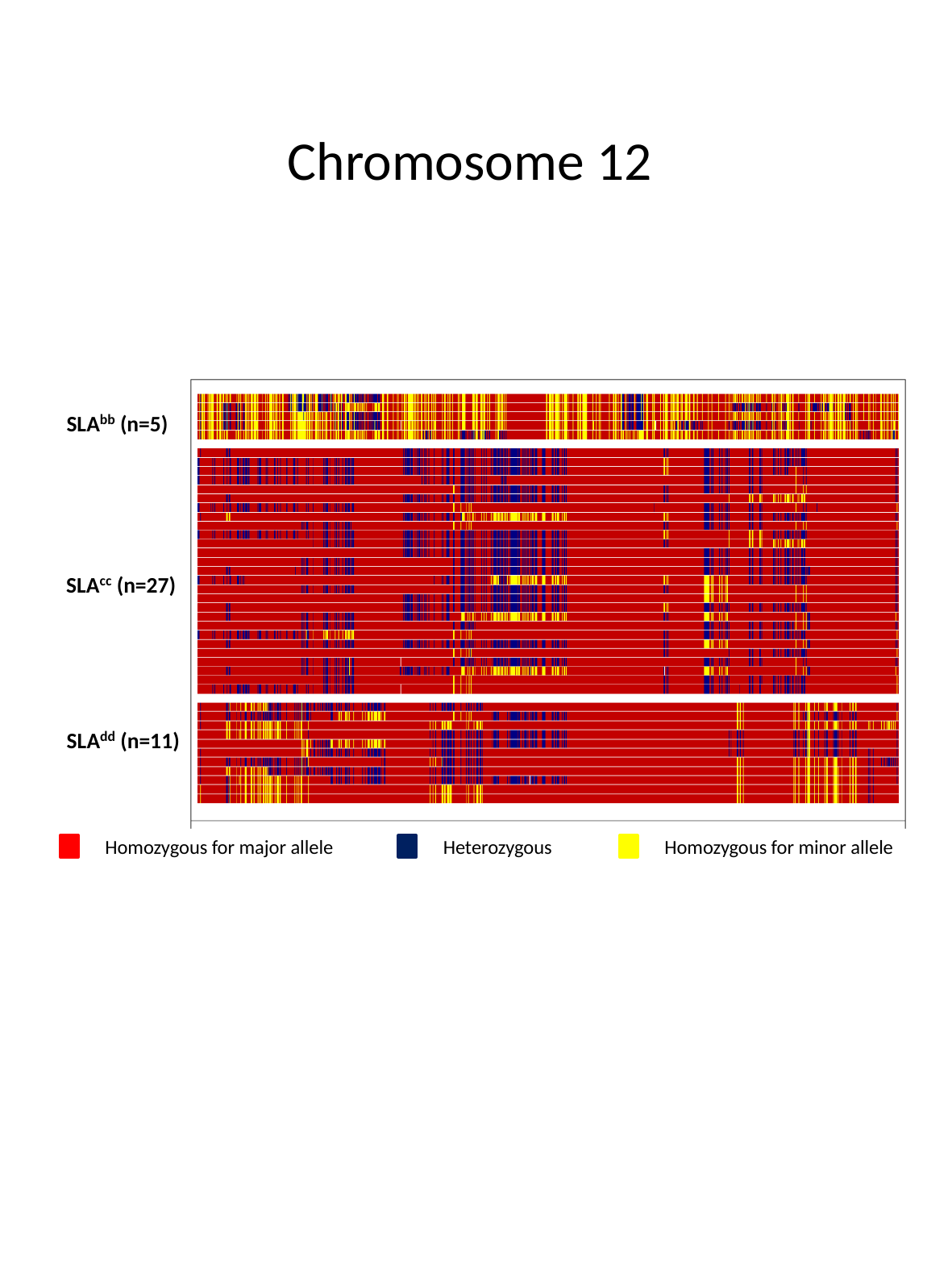

# Chromosome 12
SLAbb (n=5)
SLAcc (n=27)
SLAdd (n=11)
Homozygous for major allele
Heterozygous
Homozygous for minor allele

## Slide 14
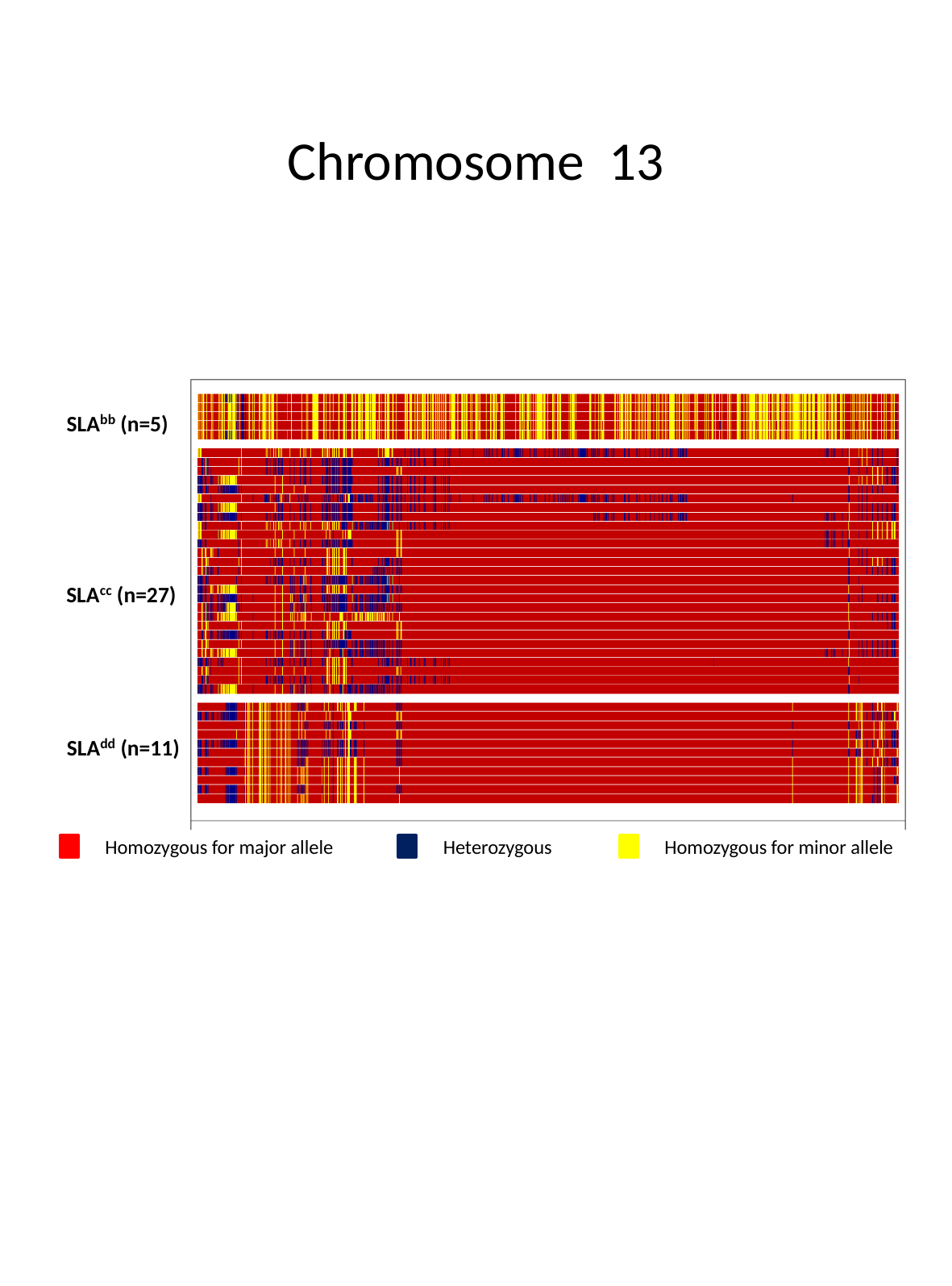

# Chromosome 13
SLAbb (n=5)
SLAcc (n=27)
SLAdd (n=11)
Homozygous for major allele
Heterozygous
Homozygous for minor allele

## Slide 15
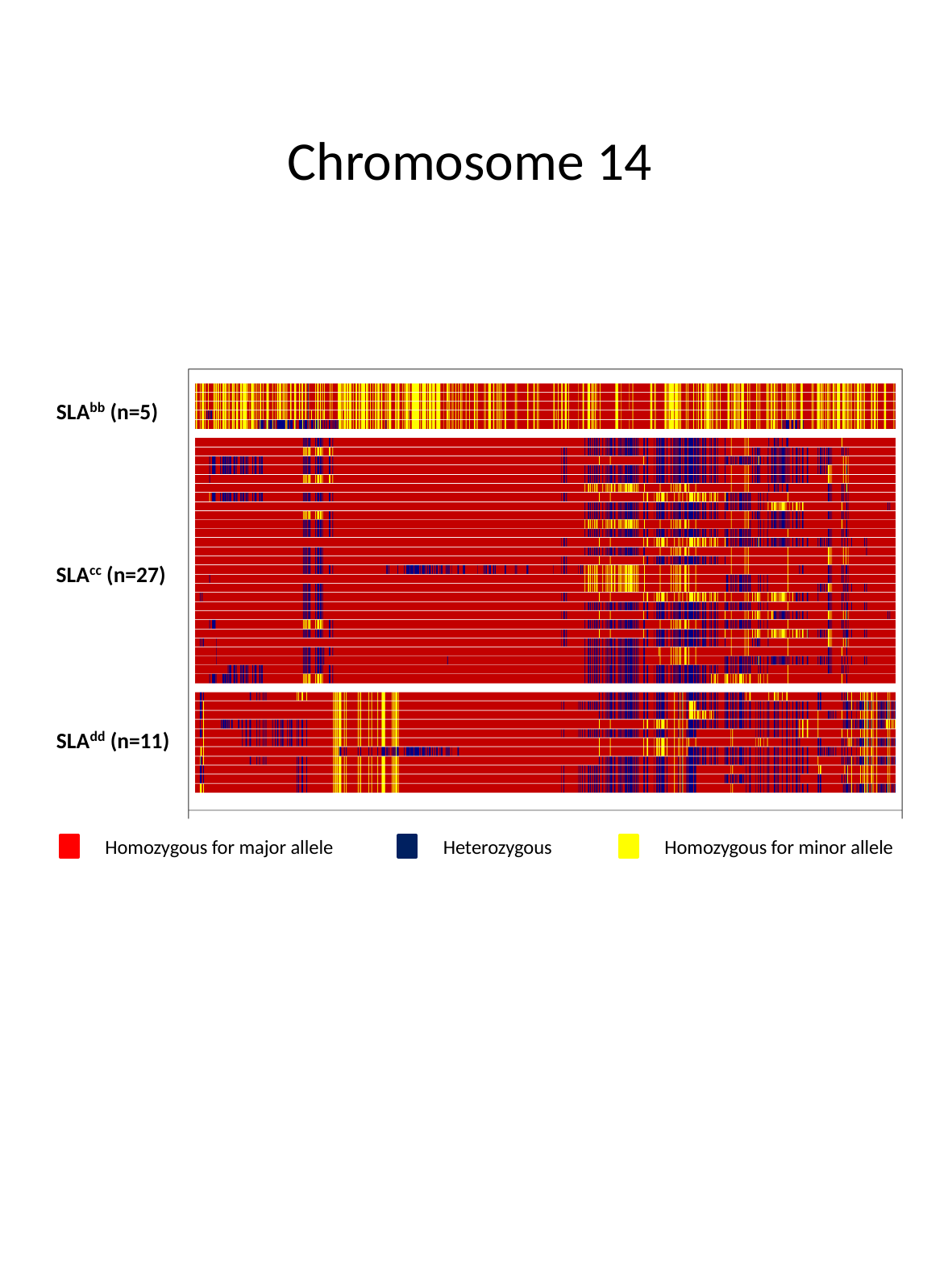

# Chromosome 14
SLAbb (n=5)
SLAcc (n=27)
SLAdd (n=11)
Homozygous for major allele
Heterozygous
Homozygous for minor allele

## Slide 16
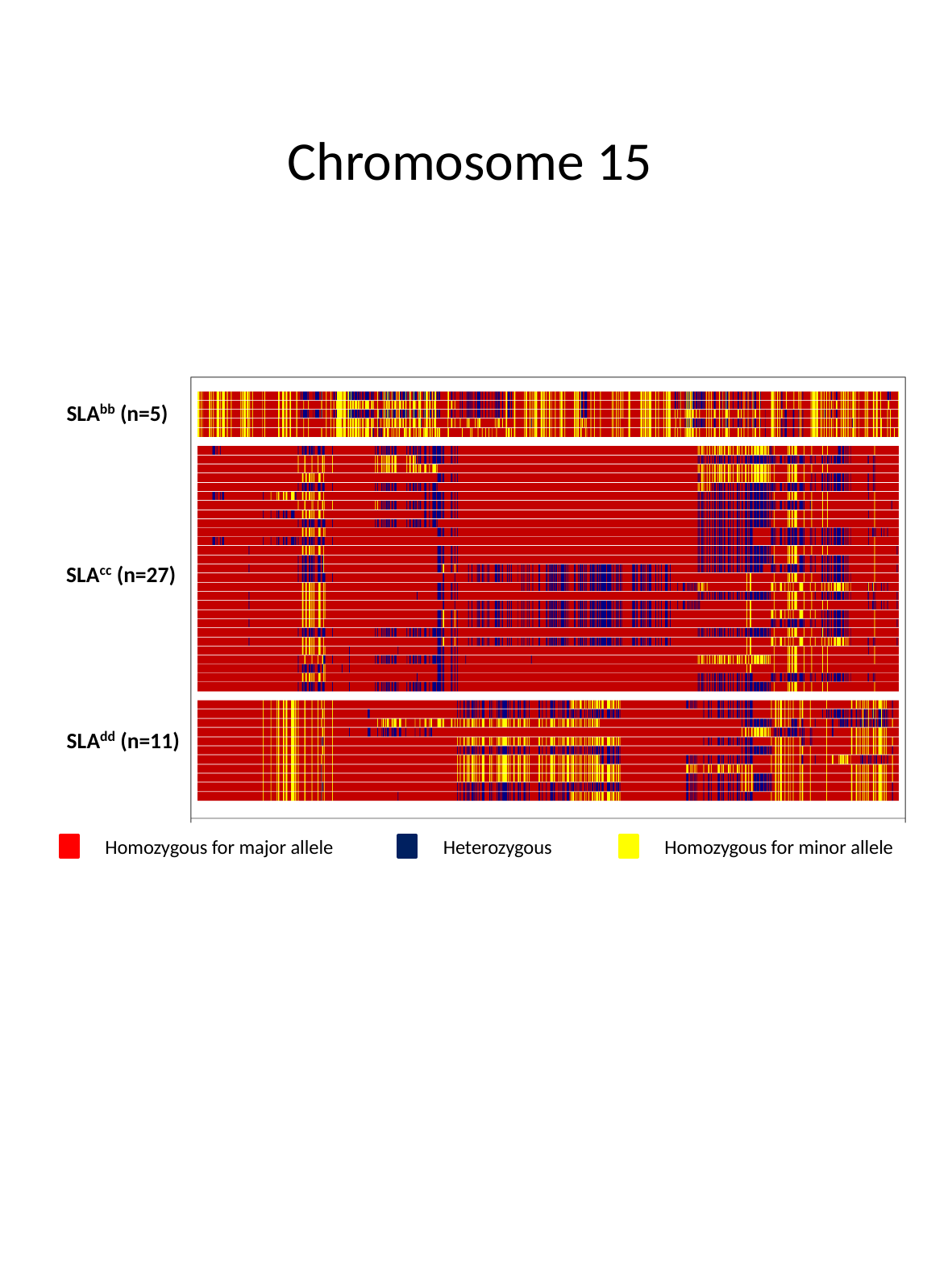

# Chromosome 15
SLAbb (n=5)
SLAcc (n=27)
SLAdd (n=11)
Homozygous for major allele
Heterozygous
Homozygous for minor allele

## Slide 17
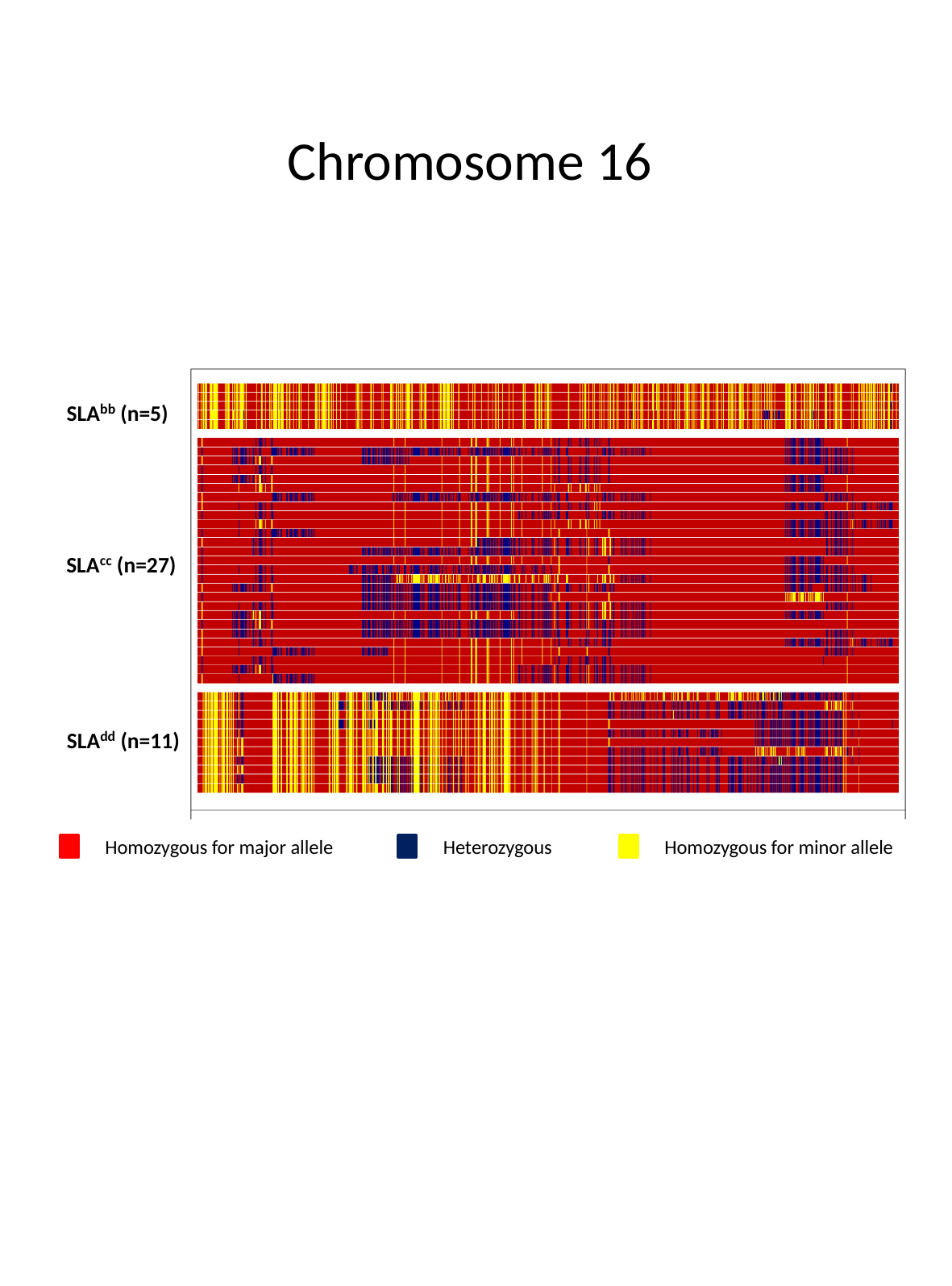

# Chromosome 16
SLAbb (n=5)
SLAcc (n=27)
SLAdd (n=11)
Homozygous for major allele
Heterozygous
Homozygous for minor allele

## Slide 18
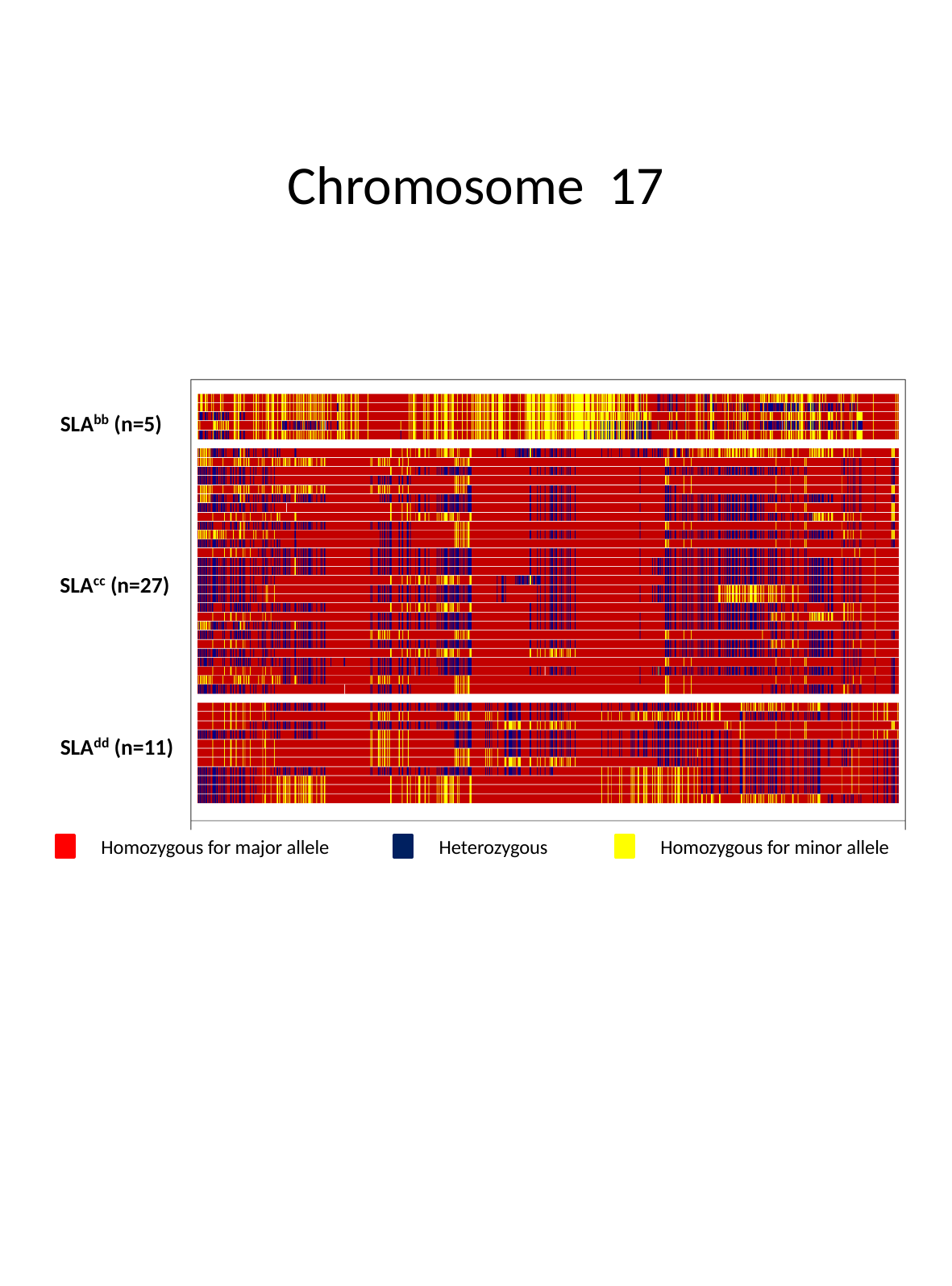

# Chromosome 17
SLAbb (n=5)
SLAcc (n=27)
SLAdd (n=11)
Homozygous for major allele
Heterozygous
Homozygous for minor allele

## Slide 19
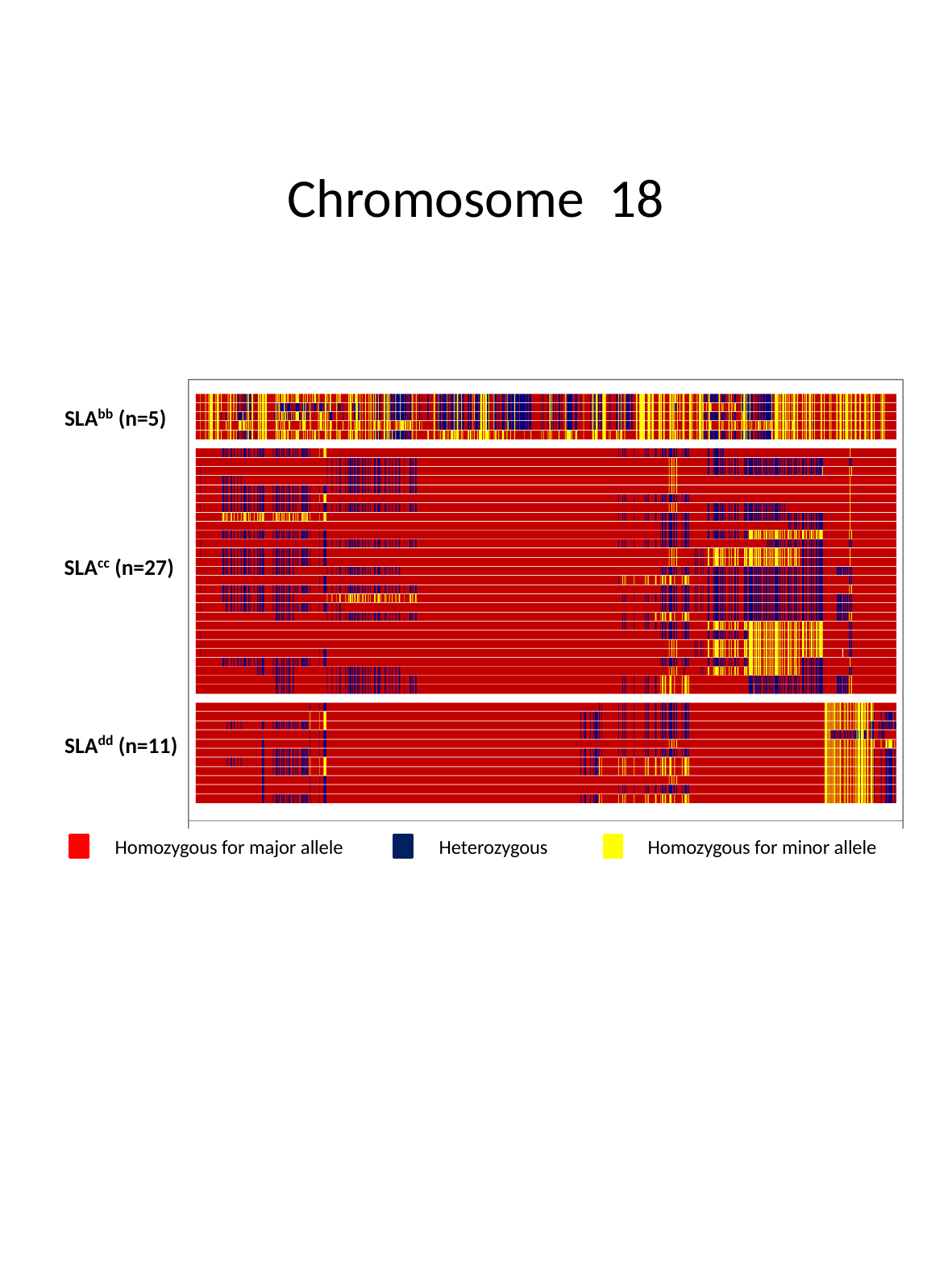

# Chromosome 18
SLAbb (n=5)
SLAcc (n=27)
SLAdd (n=11)
Homozygous for major allele
Heterozygous
Homozygous for minor allele
